# GATA3, HDAC6, and BCL6 Regulate FOXP3+ Treg Plasticity and Determine Treg Conversion into Either Novel Antigen-Presenting Cell-Like Treg or Th1-Treg

**DOI:** 10.3389/fimmu.2018.00045

**Published:** 2018-01-26

**Authors:** Keman Xu, William Y. Yang, Gayani Kanchana Nanayakkara, Ying Shao, Fan Yang, Wenhui Hu, Eric T. Choi, Hong Wang, Xiaofeng Yang

**Affiliations:** ^1^Center for Metabolic Disease Research, Lewis Katz School of Medicine at Temple University, Philadelphia, PA, United States; ^2^Center for Cardiovascular Research & Thrombosis Research, Lewis Katz School of Medicine at Temple University, Philadelphia, PA, United States; ^3^Department of Pathology, Lewis Katz School of Medicine at Temple University, Philadelphia, PA, United States; ^4^Department of Surgery, Lewis Katz School of Medicine at Temple University, Philadelphia, PA, United States; ^5^Department of Pharmacology, Microbiology and Immunology, Lewis Katz School of Medicine at Temple University, Philadelphia, PA, United States

**Keywords:** CD4+ T helper subset differentiation, CD4+ FOXP3+ regulatory T cells, metabolic cardiovascular diseases, Th1-like Treg, APC-like Treg

## Abstract

We conducted an experimental database analysis to determine the expression of 61 CD4+ Th subset regulators in human and murine tissues, cells, and in T-regulatory cells (Treg) in physiological and pathological conditions. We made the following significant findings: (1) adipose tissues of diabetic patients with insulin resistance upregulated various Th effector subset regulators; (2) in skin biopsy from patients with psoriasis, and in blood cells from patients with lupus, effector Th subset regulators were more upregulated than downregulated; (3) in rosiglitazone induced failing hearts in ApoE-deficient (KO) mice, various Th subset regulators were upregulated rather than downregulated; (4) aortic endothelial cells activated by proatherogenic stimuli secrete several Th subset-promoting cytokines; (5) in Treg from follicular Th (Tfh)-transcription factor (TF) Bcl6 KO mice, various Th subset regulators were upregulated; whereas in Treg from Th2-TF GATA3 KO mice and HDAC6 KO mice, various Th subset regulators were downregulated, suggesting that Bcl6 inhibits, GATA3 and HDAC6 promote, Treg plasticity; and (6) GATA3 KO, and Bcl6 KO Treg upregulated MHC II molecules and T cell co-stimulation receptors, suggesting that GATA3 and BCL6 inhibit Treg from becoming novel APC-Treg. Our data implies that while HDAC6 and Bcl6 are important regulators of Treg plasticity, GATA3 determine the fate of plastic Tregby controlling whether it will convert in to either Th1-Treg or APC-T-reg. Our results have provided novel insights on Treg plasticity into APC-Treg and Th1-Treg, and new therapeutic targets in metabolic diseases, autoimmune diseases, and inflammatory disorders.

## Introduction

CD4+ FOXP3+ (forkhead box P3) regulatory T cells (Treg) are classified as a subset of CD4+ T cells (T helper cells—Th), and they are specialized in suppression of immunopathological reactions in host immune system against antigens and dangers (1). In addition to inhibition of adaptive immune response, Treg also play a critical role in controlling various innate immune responses involved in cancers (2), inflammatory diseases including cardiovascular diseases and atherosclerosis (3). Also, Treg facilitate blood flow recovery after ischemia (4), control adipose tissue inflammation, and promote muscle repair (5). Treg’s role in maintaining self-tolerance and prevention of autoimmune responses and chronic inflammation is mediated by various mechanisms such as (a) Treg killing of target cells (2); (b) modulation of target cells *via* cell–cell contact; (c) secretion of anti-inflammatory/immunosuppressive cytokines (6) including interleukin-10 (IL-10), IL-35 (7–9), and transforming growth factor-β (TGF-β); as well as (d) inhibition of target cells by exosome-carried microRNAs (1). We previously reported that Treg cell death pathways (1, 10–18), Treg generated IL-35 (7–9), and epigenetic pathways (19, 20) may be novel therapeutic targets for maintaining Treg survival, preventing Treg from becoming pathological Treg (1), and suppressing vascular inflammation (3).

Current understanding on Th differentiation is that in response to stimulation by several different inducing cytokines such as interferon-γ (IFN-γ), IL-12, and IL-4, and also depending on the anatomical location (21), naïve CD4+ T cells can be differentiated/polarized into at least nine terminally differentiated Th cell subsets. These subsets include T helper cell 1 (Th1), Th2, Th9, follicular T (Tfh) (21), Th17, Treg, Th22 (1, 22), Th25 (23), and CD4+ cytotoxic T cells (CD4+ CTL) (24). Recently, we proposed a novel concept which suggests that pathological conditions re-shape physiological Treg into pathological Treg that have weakened immunosuppressive functions and increased plasticity (1). The following supporting evidence published by other investigators validate our proposed model: first, recent reports have identified Th1-like Treg phenotype in several pathological environments (25). For example, atherosclerosis-driven Treg plasticity leads to formation of a dysfunctional subset of IFN-γ secreting Th1-like Treg (26). In addition, presence of pro-inflammatory IL-17A cytokine secreting Treg had been reported (27); second, myocardial infarction increases Treg but their functions are compromised (28). This is an indication that Treg are converted to pathological Treg and can become less suppressive under pathological conditions; third, lymphomas “push” physiological Treg into four different types of “lymphoma Treg” (2); fourth, self-reactive T cells, termed anti-Treg, that can recognize MHC class I-restricted antigen peptide epitopes derived from Treg markers (such as indoleamine 2,3-dioxygenase (IDO), tryptophan 2,6-dioxygenase (TDO), programmed death ligand 1 (PDL1), and forkhead box P3 (FOXP3)) were identified. This is a clear indication that there are endogenous mechanisms available to suppress Treg under various pathologies (29); and fifth, a recent report showed that a Treg transcription factor FOXO3 is highly expressed in tolerogenic dendritic cells (DCs) and program their tolerogenic influence, which modulate Treg and activate anti-Treg (30). It is accepted that Treg undergo phenotypic, and functional plastic changes into other Th subsets under pathological conditions (22, 31). However, the issue of whether Treg convert into other immune cell types such as APCs (antigen-presenting cells) when given the right condition remains unknown.

**Figure 1:**
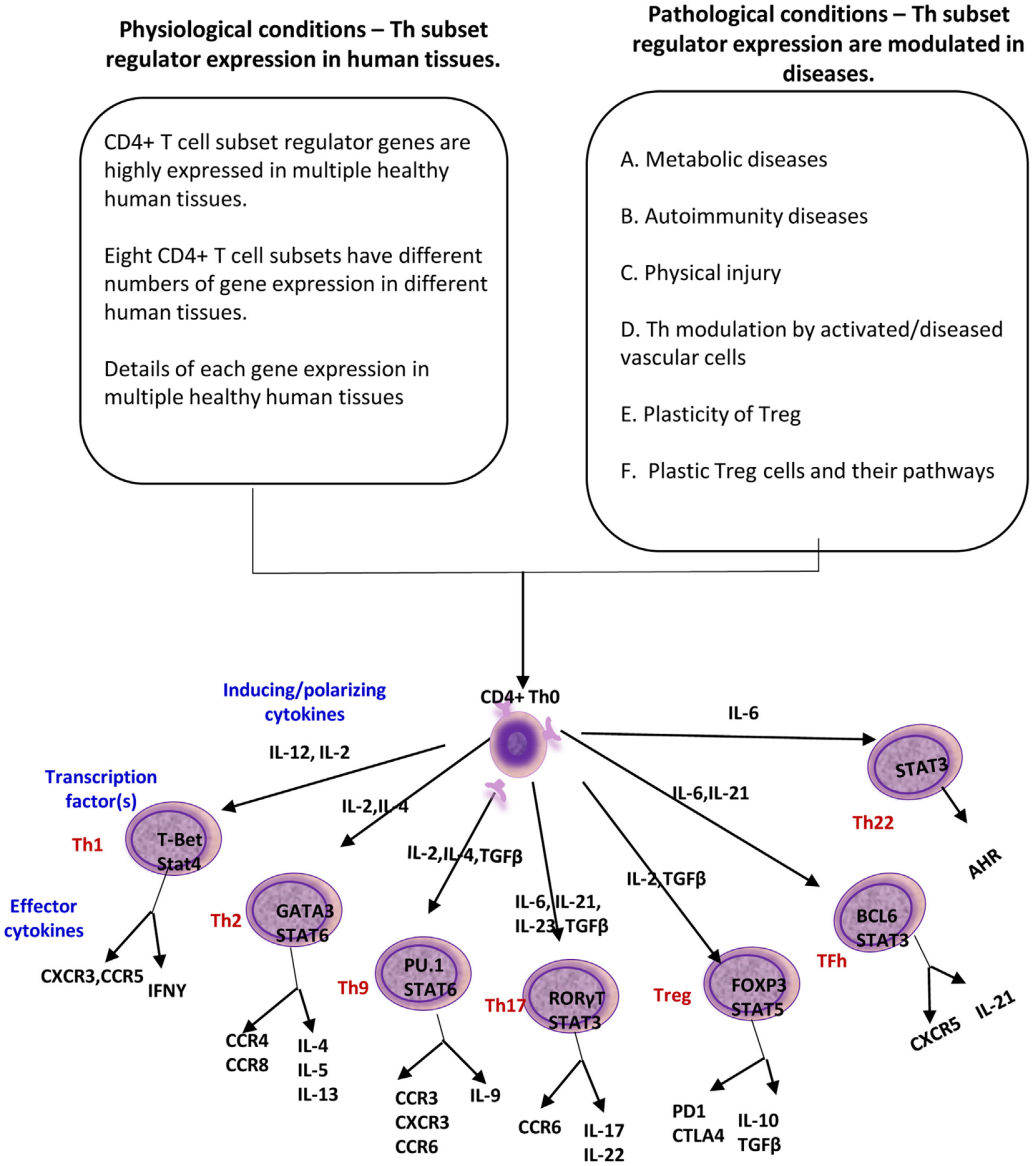
A novel working hypothesis: CD4+ T helper (Th) subset regulators are differentially expressed in normal tissues in physiological conditions; and Th subset regulator expression are modulated in various diseases and mutant mice (Th25 information is limited; therefore, Th25 is not included in this figure).

Despite recent significant progress in T cell research, there are several aspects of Th subset regulator expression that have not yet been explored: first, the expression profiles of all the Th subset regulators in tissues under physiological and pathological conditions, specifically in patients with metabolic cardiovascular diseases have not been studied; second, the issue of whether the expression of Th subset-promoting cytokines are modulated in vascular cells in response to pathological stress is not known; third, mechanistically, the issue of whether the master regulators of other Th subsets can control Treg plasticity had been poorly characterized; and fourth, whether plastic Treg can function as APCs is yet to be determined. To address these questions, we took a “panoramic view” at the tissue expression patterns of 61 identified Th subset regulators. Our novel working hypothesis is presented in Figure 1. Our results demonstrated that all the Th subset regulators are differentially expressed among tissues in physiological conditions. We also found that the expression of other Th subset master regulators control Treg plasticity to Th1 and APC-like Treg. Our findings provide novel insights into Th subset master regulators such as FOXP3, GATA3, BCL6, and HDAC6, suggesting that these signaling molecules can be potential therapeutic targets in the treatment of cancers, metabolic diseases, inflammation, tissue regeneration, and repair.

## Materials and Methods

### Tissue Expression Profiles of Genes Encoding Th Subset Regulators

**Figure 2:**
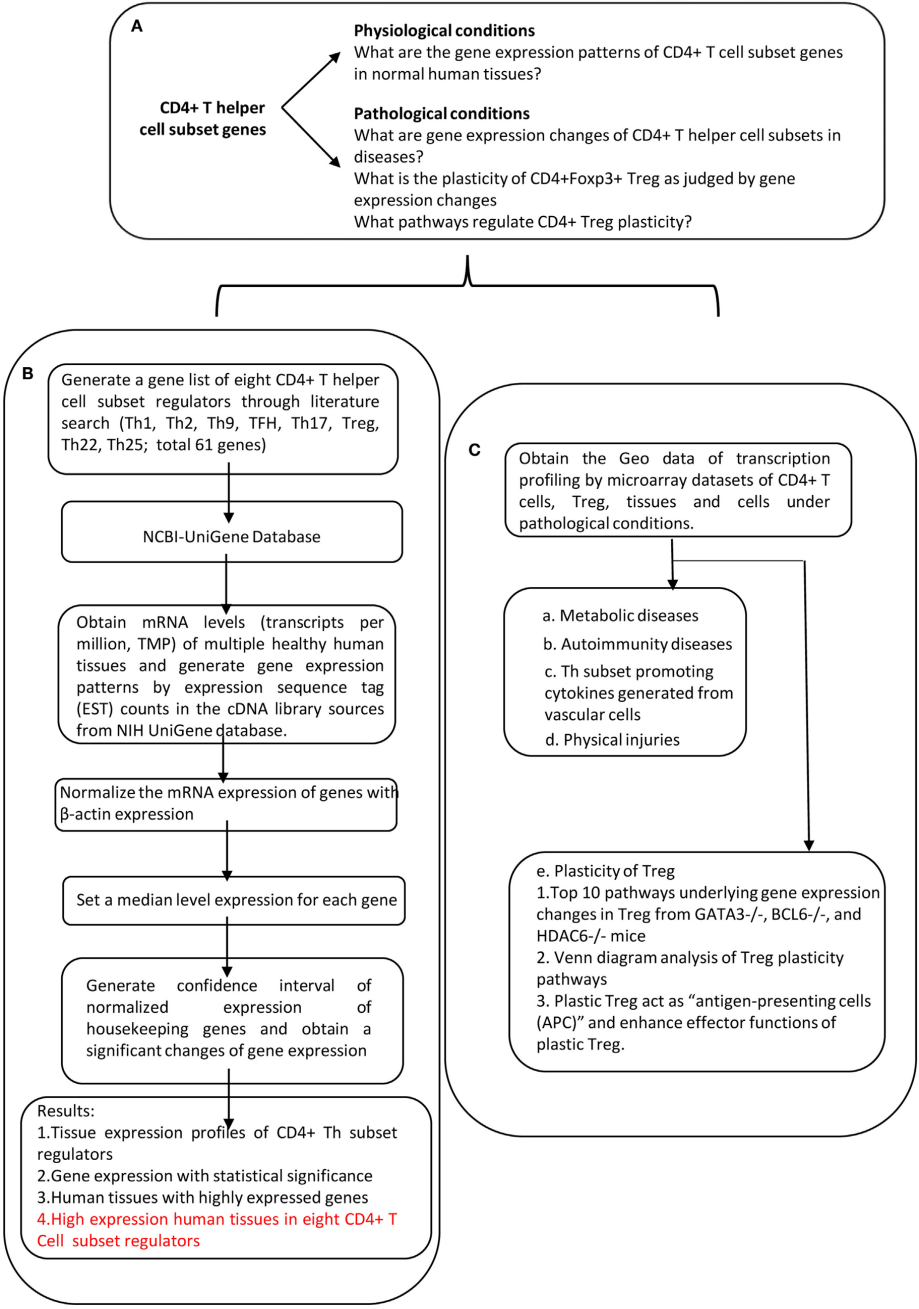
Flow chart of database mining strategy and two parts of data organization. **(A)** Presents the key questions addressed by this study. **(B)** Determining the expression of Th subset regulators in tissues under physiological conditions. **(C)** Determining the expression of Th subset regulators under pathological conditions.

An experimental data mining strategy (Figure 2) was used to analyze the expression profiles of mRNA transcripts of Th subset regulator genes in 32 human tissues including heart and vasculature. We utilized experimentally verified mRNA expression in the EST (expressed sequence tag) databases of the National Institutes of Health (NIH)/National Center of Biotechnology Information (NCBI) UniGene (http://www.ncbi.nlm.nih.gov/sites/entrez?db=unigene) to determine the transcription profile of Th subset regulators in tissues of interest. Transcripts per million of genes of interest were normalized to that of housekeeping β-actin in each given tissue to calculate the arbitrary units of gene expression as we previously reported (7, 32). Confidence intervals of the expression variation of housekeeping genes were generated by calculating the mean plus two times that of the SD of the arbitrary units of three randomly selected housekeeping genes (PRS27A, GADPH, and ARHGDIA in human; Ldha, Nono, and Rpl32 in mouse) normalized by β-actin in the given tissues (Figure 2). If the expression variation of a given gene in the tissues was larger than the upper limit of the confidence interval (the mean plus two times the SD) in housekeeping genes, the high expression levels of genes in the tissues were considered statistically significant. Gene transcripts where the expression level was lower than one per million were technically considered as no expression.

### Expression Profiles of Th Subset Regulators in Disease Models and Cell Activity

Microarray datasets were collected from the NIH-NCBI-Geo DataSets (https://www.ncbi.nlm.nih.gov/gds/). These data included the information of the expression level of Th subset regulators through experiments submitted directly to the NCBI Gene Expression Omnibus database. We used the gene expression data from the following microarray experimental settings: (1) metabolic diseases such as obesity (33), type 2 diabetes (34), failing heart (35), hyperlipidemic apolipoprotein E-deficient (ApoE−/−) mice (36), and familial hypercholesterolemia (37); (2) autoimmune diseases such as systemic lupus erythematosus (38) and psoriasis (39); (3) severe trauma (40) and burn injury (41); (4) vascular endothelial cells and vascular smooth muscle cells stimulated with proatherogenic stimuli; and (5) Treg in diseased and gene mutation conditions.

## Results

### All the Th Subset Regulators Are Differentially Expressed in Various Human Tissues Including Heart and Vascular Tissues in Physiological Conditions

In response to the stimulation by several different inducing cytokines such as interferon-γ (IFN-γ), IL-12, and IL-4, naïve CD4+ T cells can be differentiated/polarized into at least eight terminally differentiated subsets including T helper cell 1 (Th1), Th2, Th9, follicular T (Tfh), Th17, Treg, Th22 (1, 22), and Th25 (23) (Figure 1). An important question remains whether human tissues provide various stimulatory/suppressive environments that differentially regulate Th subset polarization/differentiation. We hypothesized that differentiation signals and immunogenic conditions of tissues regulate the expression of Th subset regulators in human tissues. To examine this hypothesis, we conducted an extensive literature survey (22, 23, 42) and collected total of 61 Th subset regulators (Table 1). These Th subset regulators included 20 Th subset transcription factors, 5 transcription activators, 1 transcription repressor, 2 signal transducers and transcription modulators, 20 cytokines, 5 lineage inducing factors, 6 cytokine/factor receptors, and 2 other membrane receptors (Table 1). We then examined the expression patterns of all 61 Th subset regulators in 32 human tissues as described in the Section “Materials and Methods.”

**Table 1:** 61 Genes involved in regulation of eight CD4+ T helper cell (Th) subsets that were focused in our study.

Gene name	Common name	UniGene ID (Human)	UniGene ID (mouse)	Th Subset
**Transcription factor (20)**				
AHR	Aryl hydrocarbon receptor	Hs.171189	Mm.341377	TH22
ACT1	Act1	N/A	N/A	TH25
BATF	BATF	Hs.509964	Mm.6672	TFH
BACH2	BACH2	Hs.269764	Mm.491223	Treg
BCL6	BCL6	Hs.478588	Mm.347398	TFH
CMIP	c-MAF	Hs.594095	Mm.233181	TH2
EOMES	EOMES	Hs.591663	Mm.200692	TH1
FOXO1	FOXO1	Hs.370666	Mm.29891	Treg
FOXO3	FOXO3	Hs.220950	Mm.338613	Treg
FOXP3	Foxp3	Hs.247700	Mm.182291	Treg
GATA3	Gata-3	Hs.524134	Mm.313866	TH2
HNF1A	TCF1	Hs.654455	Mm.332607	TFH, TH17
IRF4	IRF4	Hs.401013	Mm.4677	TH2, TH9, TFH
IKZF4	IKZF4	Hs.553221	Mm.358648	Treg
MAF	MAF	Hs.134859	Mm.439772	TFH
RORC	RORυT	Hs.256022	Mm.4372	TH17
RORA	RORα	Hs.560343/Hs.655155	Mm.427266	TH17
RUNX3	RUNX3	Hs.170019	Mm.378894	TH1
SPI1	PU.1	Hs.502511	Mm.1302	TH9
TBX21	T-bet	Hs.272409	Mm.94519	TH1

**Transcription activator (5)**				
STAT1	STAT1	Hs.743244	Mm.277406	TH1
STAT3	STAT3	Hs.463059	Mm.249934	TFH
STAT4	STAT4	Hs.80642	Mm.1550	TH1
STAT5A	STAT5	Hs.437058	N/A	Treg
STAT6	STAT6	Hs.524518	Mm.121721	TH9

**Transcription repressor (1)**				
GFI1	GFI1	Hs.73172	Mm.2078/Mm.453139	TH2

**Signal transducers and transcriptional modulators (2)**				
SMAD2	SMAD2	Hs.12253	Mm.152699	TH9, Treg
SMAD3	SMAD3	Hs.727986	Mm.7320	TH9, Treg

**Cytokine (20)**				
CCL15	CCL15	Hs.272493	N/A	TH22
CCL17	CCL17	Hs.546294	Mm.41988	TH22
IL2	IL-2	Hs.89679	Mm.14190	TH1, TH2, TH9, Treg
IL4	IL-4	Hs.73917	Mm.276360	TH2, TH9
IL5	IL-5	Hs.2247	Mm.4461	TH2, TH9
IL6	IL-6	Hs.654458	Mm.1019	TFH, TH17
IL9	IL-9	Hs.960	Mm.3006	TH9
IL10	IL-10	Hs.193717	Mm.874	Treg
IL12RB1*	IL-12	Hs.567294	N/A	TH1
IL13	IL-13	Hs.845	Mm.1284	TH2
IL17B	IL-17	Hs.156979	N/A	TH17
IL17F	IL-17F	Hs.272295	Mm.222807	TH17
IL21	IL-21	Hs.567559	Mm.157689	TFH, TH17
IL22	IL-22	Hs.287369	Mm.103585	TH17
IL23	IL-23	N/A	N/A	TH17
IL25	IL-25	Hs.302036	Mm.90154	TH25
IL27	IL-27	Hs.528111	Mm.222632	TH1
IL10RB	IL10Rβ	Hs.654593	Mm.4154	TH22
ICOS	ICOS	Hs.56247	Mm.42044	TFH
IFNG	IFNγ	Hs.856	Mm.240327	TH1

**Factors inducing lineage (5)**				
CXCR5	CXCR5	Hs.113916	Mm.6246	TFH
IDO1	Indoleamine 2,3-dioxygenase 1	Hs.840	Mm.392	TH2
MYD88	MyD88	Hs.82116	Mm.213003	TH17
MTOR	mTOR	Hs.338207	Mm.21158	Treg
TGIF1	TGFβ	Hs.373550	Mm.101034	TH9, TH17, Treg

**Receptor (6)**				
RARA	RARA	Hs.654583	Mm.439744	TH1
CCR3	CCR3	Hs.506190	Mm.57050	TH9
CCR4	CCR4	Hs.184926	Mm.1337	TH2
CXCR3	CXCR3	Hs.198252	Mm.12876	TH1, TH9
CCR6	CCR6	Hs.46468	Mm.8007	TH17
CCR8	CCR8	Hs.113222	Mm.442098	TH2

**Others (2)**				
CD28	CD28	Hs.443123	Mm.255003	Treg
CTLA4	CTLA4	Hs.247824	Mm.390	Treg

*^a^Hemopoietin receptor*.

*The Information in this table were collected from the three references indicated (22, 23, 42)*.

Based on the Th subset regulator expression amongst human tissues examined, we classified the tissues into following three groups: highly expressed (++), low expressed (+), and not expressed (−) as summarized in Table 2. Our analysis indicated that Th subset regulators are differentially expressed in 32 human tissues in physiological conditions (Figure S1 in Supplementary Material). Interestingly, Treg-specific transcription factor (TF) FOXP3 was expressed in trachea, thymus, spleen, mammary gland, lymph node, lung, eye, and blood. Out of these tissues, trachea highly expressed FOXP3 TF. Th1 TF TBX21 (T-bet) was only expressed in spleen, lymph nodes, and pancreas with the highest level recorded in spleen. Th2 TF GATA3 was expressed in 21 out of 32 tissues that were analyzed, including heart and vasculature with the high levels in adrenal gland, mammary gland, and placenta. Moreover, Th9 TF SPI1 (Pu.1) was expressed in 16 tissues with high expression levels in 11 tissues. Tfh TF BCL6 was expressed in 28 tissues with the high levels in adipose tissue, nerve, and trachea. Additionally, Th17 TF RORγt was expressed in 18 tissues with the high levels in bladder, cervix, liver, lymph node, muscle, thymus, and trachea. Th22 transcription regulator STAT3 was expressed in 31 tissues. Of note, the regulators of Th25 subset are unavailable for examination in this study. CD4 CTL are found within the traditional classification of Th0, Th1, Th2, TH17, CD4 intraepithelial lymphocyte (IEL) subsets with the characteristic expression of class I-restricted T cell associated molecule (CRTAM+) (24), which is outside of the scope of this study.

**Table 2:** The expression of Th subset regulators are differentially expressed in human tissues^a^.

Gene/tissue	Adipose tissue	Adrenal gland	Bladder	Blood	Bone	Bone marrow	Brain	Cervix	Embryonic tissue	Esophagus	Eye	Heart	Intestine	Kidney	Liver	Lung	Lymph node	Mammary gland	Muscle	Nerve	Ovary	Pancreas	Placenta	Prostrate	Skin	Spleen	Stomach	Thymus	Trachea	Umbilic chord	Uterus	Vascular
AHR	+		+	+	+	+	+		+	+	+	+	+	+	+	+	+	+	++	++		+	++	+	+		+	+	+	+	+	+
BATF				+									+			+	++	+						+	+		+				+	+
BACH2				++			++		+		+					+	++	+	++			++		+				+				
BCL6 (Tfh*)	++	+	+	+	+		+	+	+		+	+	+	+	+	+	+	+	+	++	+	+	+	+	+	+	+	+	++		+	+
CMIP	+	+	++	+	++		+		+		++	+	+	+	+	++	+	+	+	++	+	+	+	+	+	+	+	+	++		+	+
EOMES							+		+		+					+		+													+	
FOXO1				+	+	+	+	+	+		+	+	+	+	+	+	++	+	+	+	++	+	+	+	+		+	+	+		+	+
FOXO3 (Treg)	+	+		+	+		+	+	+	+	++	+	+	+	+	+	+	+	++	+	+	+	+	+	+	+	+	+	+	+	+	+
GATA3 (Th2)		+	++	+			+	+	+		+	+		+	+	+	+	++		+			++	+	+		+	+			+	+
HNF1A											+		+	+	+ +												+					
IRF4				++			+				+	+	+	+		+	++			+			+	+	++	+	++	+			+	
IKZF4				+	+		+		+		++	+	+	+	+	+	+	+	+		+	+	+		+			+			+	
MAF	+	+		+	+		+	+	+		+	+	+	++	+	+	+	+	++	++	+	+	+	+	+	+	+				+	+
RORC (Th17)		+	++		+		+	++					+	+	++	+	++	+	++			+		+			+	++	++		+	
RORA		++			+		+				++		+	++	++	+		++	++		+	++	++	+	++		+	+	++		+	+
RUNX3	+			+	+	+	+	+	+		+	++	+	+		+	++	+				+	+	+	+	++	+	+	++		+	
SPI1 (Th9)				++			+				+	++	++	++	++	++	++	+			++		++	+		++	+	++				
TBX21 (Thl)																	+					+				++						
STAT1	+	+	+	+	+	+	+	+	+		+	+	+	+	+	+	+	+	+	+	+	+	+	+	+	+	+	+	++		+	+
STAT3 (Th2)	+	+	+	+	+	+	+	+	+	+	+	+	+	+	+	+	+	+	+	++	+	+	+	+	+	+	+	+	++		+	+
STAT4				++		++	+		+		+	++	+	+		+	+	+	++		+		++		+	++		++			+	
STAT5A	+		+	+	+		+	+	+		+	+	+	+	+	+	++	+	+		+	+	+	+	+	+	+	+	++		+	+
STAT6		+	+	+	+	+	+	+	+	+	+	+	+	+	+	+	+	+	+	+	+	+	+	+	+	+	+	+	++	+	+	+
GFI1				+		++							+			+	+					+				+		+			+	
SMAD2	+	+	+	+	+	+	+	+	+		+	+	+	+	+	+	+	+	+	++	+	+	+	+	+	+	+	+			+	+
SMAD3	+	+	+	+	+	+	+	+	+	+	+	+	+	+	+	+	+	+	++		+	+	+	+	+	+	+	+	+		+	+
CCLL5							+						+	++	+	+								+					++		+	
CCL17																		+														
IL2				++																												
IL4				++																						++						
IL5				+	++		+				++		+	++		++	+		++		+	++	+		++	+		+				++
IL6	+	+	+	+	+	+	+	+	+		+	+	+	+	+	+		+	+	+	+	+	+	+	+	+	+	+	++	+	+	+
IL9														++																		
IL10				+												+																
L12RB1	++			+	+	+	+						+	+	+	+	+						+		+	++	+	+				
L13						++	+																									
IL7B					++						+	++				+							+	+								
IL17F				++																												
IL22				++																												
IL27																++																
L10RB		+		+		+	+		+	+	++	++			+	+	+	+	++	+	+	+	+	+					++	+	+	+
ICOS				+								++				+												+			+	
IFNG				++										+			+															
CXCR5				++						+			+			+	++		+					+	+	++		+				
IDO1					+				+		+	+	+	+	+	+		+	+		+	+	++				+				+	
MYD88		+	+	+	+	+	+	+	+	+	+	+	+	+	+	+	+	+	+		+	+	+	+	+	+	+	+	++	+	+	+
MTOR	+			+	+	+	+	+	+	+	+	+	+	+	+	+	++	+	++		+	+	+	+	+	+		+	++		+	+
TGIF1	+		+	+	+		+	+	+		+	+	+	+	+	+	+	+	++		+	+	+	+	+	+	+	+			+	
RARA		+		+	+	+	+	+	+	+	+	+	+	+	+	+	+	++	+	+	+	+	+	+	+	+	+	+	++		+	+
CCR3				++																								+				
CCR4	++			+												+		+														
CXCR3				+									+						++							+						
CCR6				+		+							+				+	+						+		+	+	+				
CCR8				++																												
CTLA4				+										+		+	++	+						+								

*Treg transcription factor Foxp3 is expressed in trachea, thymus, spleen, mammary gland, lymph nodes, lung, eye, and blood; but the expression of Th1 transcription factor Tbx21 is limited to spleen, lymph node, and pancreas*.

*“+” Human tissue with detectable gene expressions in the NIH-NCBI-UniGene database*.

*“++” Human tissue with high gene expressions (X + 2D > 4.45)*.

*^a^Transcription factor associated with Th subsets were highlighted with red fonts*.

**Figure 3:**
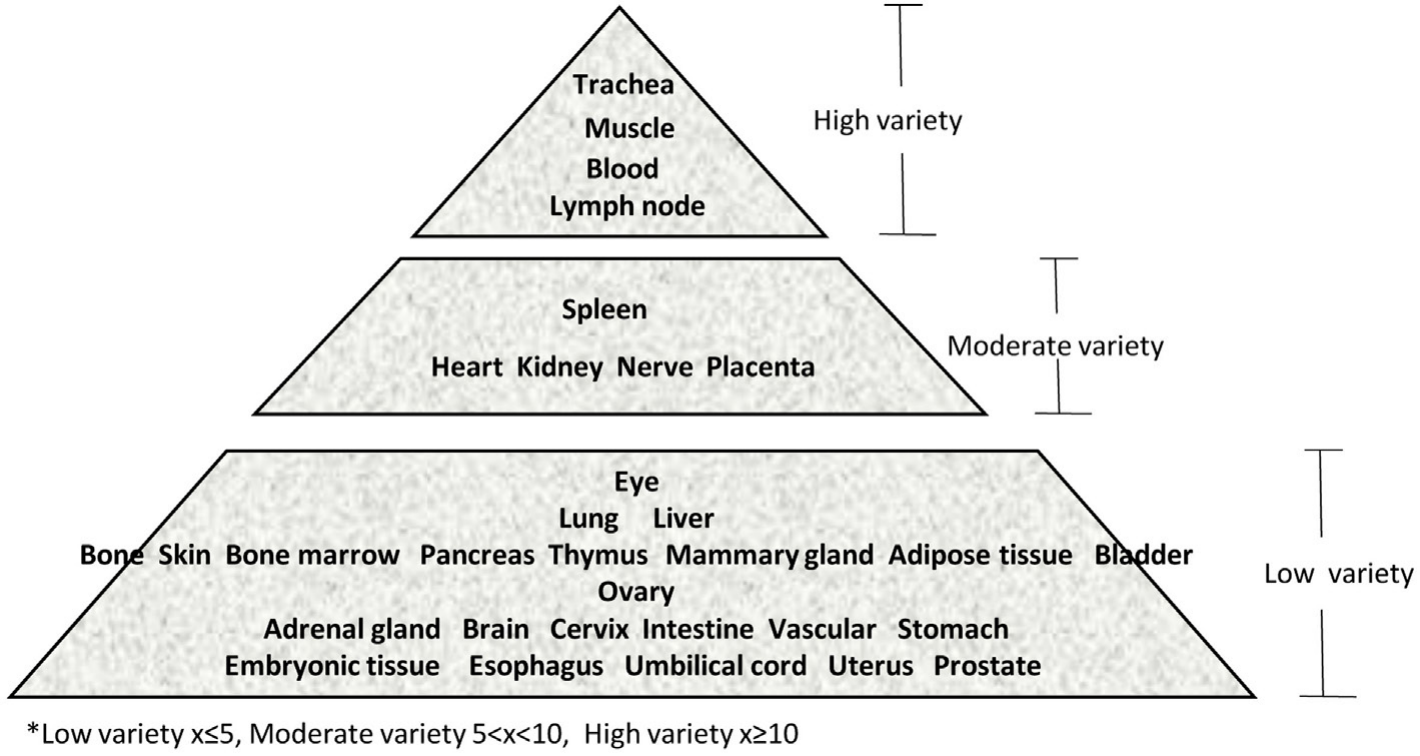
Our newly proposed “tissue pyramid” model. We constructed a tissue pyramid based on the variety of Th subset regulators expressed in tissues.

Immune privilege is the ability of tissues to actively regulate and direct immune responses that takes place in the tissue itself (43). We hypothesized that immune privilege status observed in certain human tissues may not allow Th subset regulators to be expressed. As shown in Table 3, we found that Th1 and Th2 regulators are expressed in every tissue except umbilical cord (Th1 and Th2) and esophagus (Th2). Some Th1 regulators are highly expressed in 13 human tissues including trachea, thymus, placenta, pancreas, muscle, mammary gland, lymph node, lung, heart, bone marrow, blood, and adipose tissue. In addition, we found that Th9, Tfh, Th17, and Treg regulators are expressed in every tissue (Tables 4 and 5). Th22 regulators were expressed in many tissues except spleen, cervix, adrenal gland, and adipose tissue (Tables 2–6). Moreover, based on the variety of highly expressed Th subset regulators in any given human tissues, we constructed a tissue pyramid (Figure 3). Our data indicated that trachea, muscle, blood, lymph node, spleen, heart, kidney, nerve, and placenta are located in the top of the “tissue pyramid” as they express a large variety of Th subset regulators (Figure 3).

**Table 3:** Th1 regulators are expressed in every tissue except umbilical cord; and Th2 regulators are expressed in every tissue except umbilical cord and esophagus.

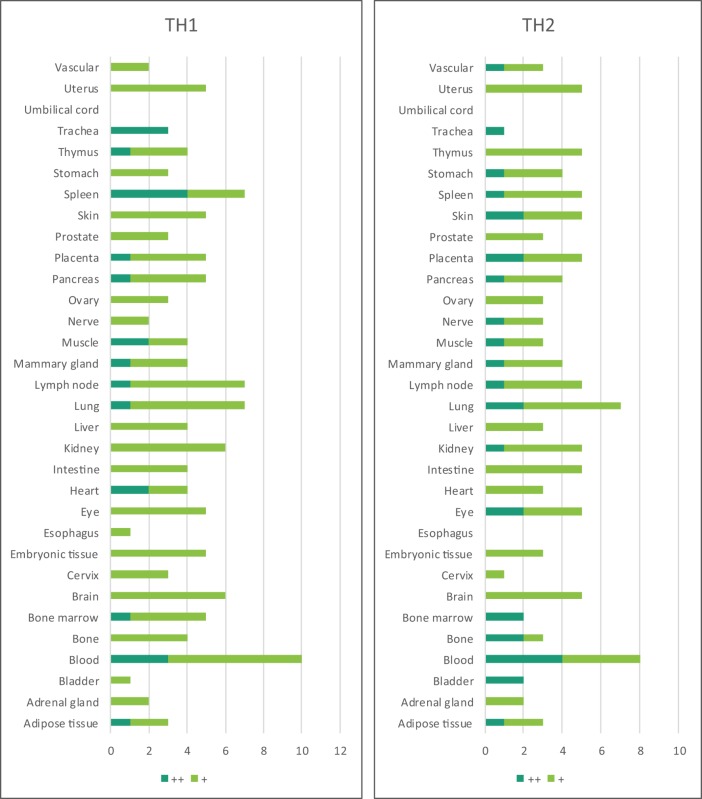

**Table 4:** Th9 regulators and Tfh regulators are ubiquitously expressed in all the human tissues examined.

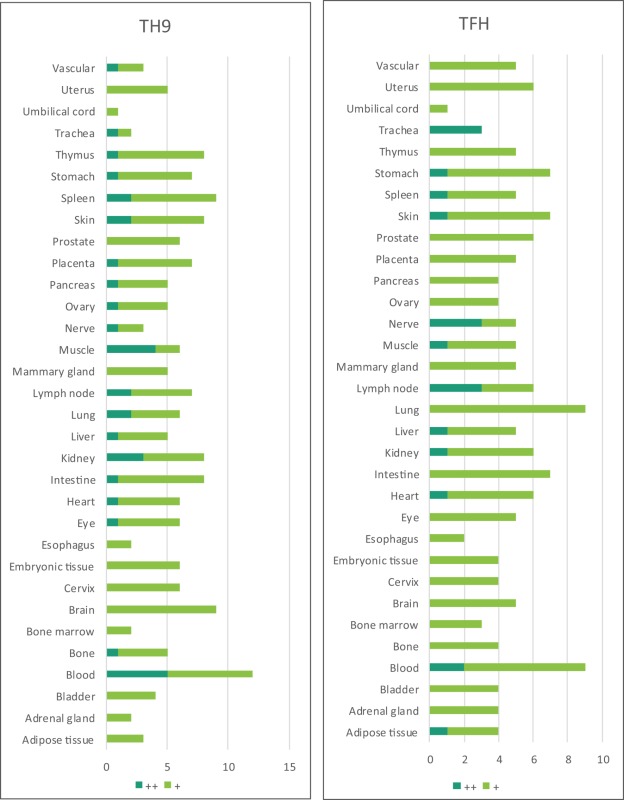

**Table 5:** Th17 regulators and T-reg regulators are ubiquitously expressed in every tissue.

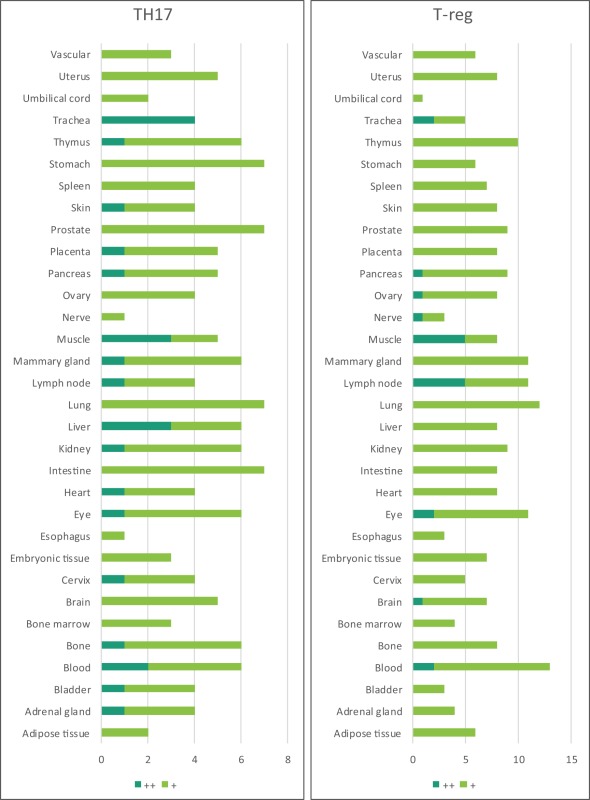

**Table 6:** Th22 regulators are expressed in every tissue except spleen, cervix, adrenal gland, and adipose tissues.

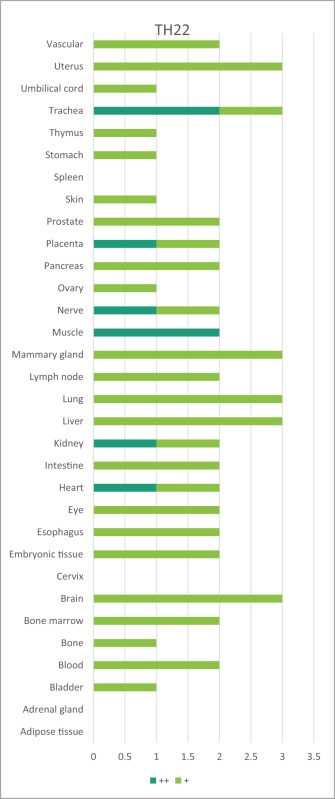

**Figure 4:**
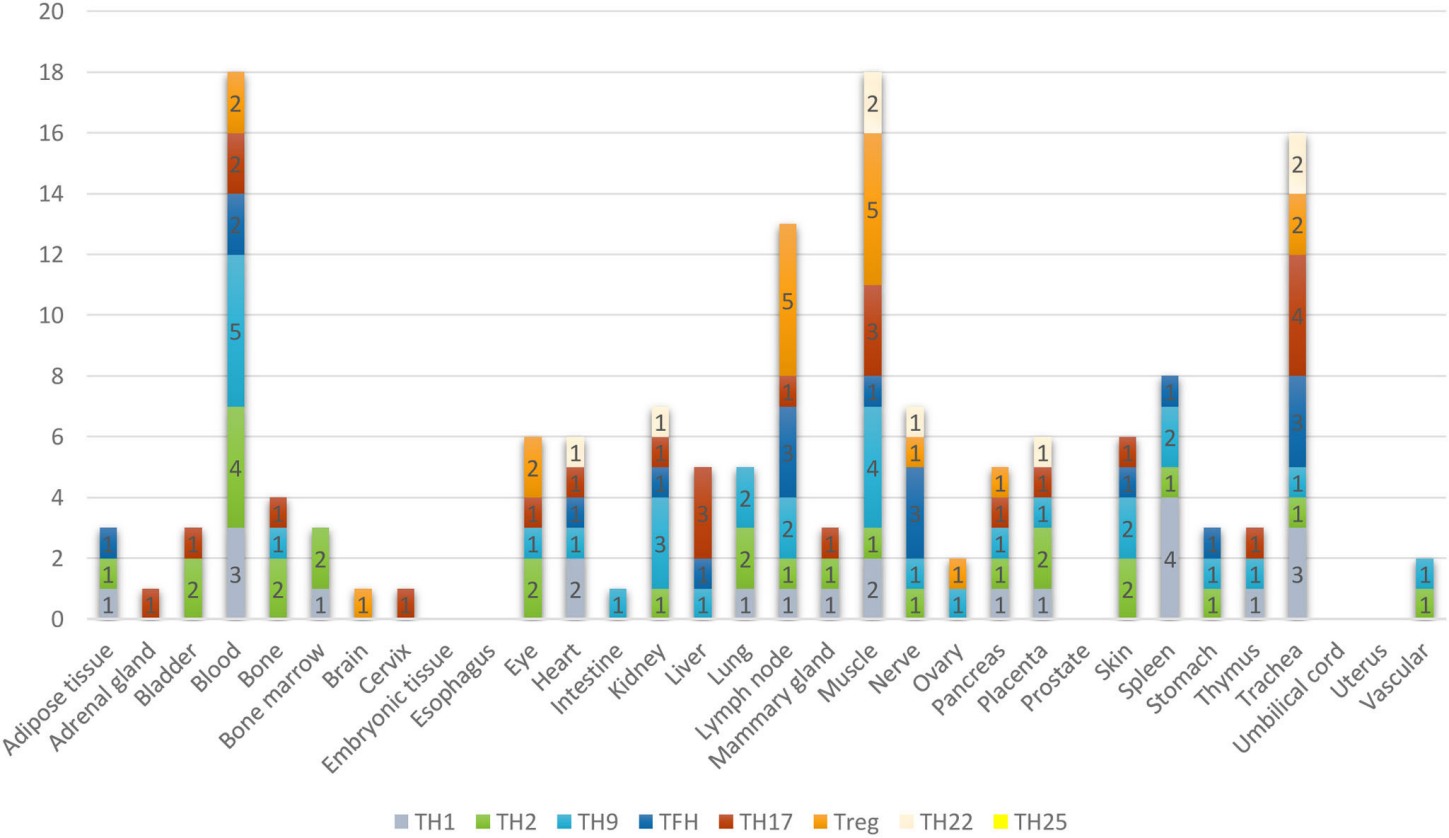
The composition of Th subsets in human tissues are different. Treg regulators are dominant in brain, eye, lymph nodes, and muscle: Th17 regulators are dominant in adrenal gland, cervix, umbilical cord, and uterus have no physiologically resident Th activities.

Furthermore, based on the relative numbers of each Th subset regulators expressed in human tissues, we discovered the following (Figure 4): (a) Treg regulators are highly expressed in brain, eye, lymph node, and muscle. (b) Th17 regulators are dominant in adrenal gland, cervix, liver, and trachea. (c) Th1 regulators are dominant in heart and spleen. (d) Th2 regulators are dominant in adipose tissue, bladder, bone marrow, eye, lung, placenta, skin, and vascular, which functionally relate to their allergy activities; (e) Tfh regulators are dominant in adipose tissue, intestine, and nerve. Of note, it was reported that Tfh is able to convert into Tfh1 and Tfh17. Future studies are needed to determine the tissue locations of those plastic Tfh subsets in addition to the classical tissue locations within and in proximity to germinal centers in secondary lymphoid organs and circulating blood (44). (f) Five human tissues including embryonic tissue, esophagus, prostate, umbilical cord, and uterus have either no or very low resident Th subset activities, which suggest that these tissues may have immune privilege status (32). Finally, (g) we found that four human tissues including blood, lymph node, muscle, and trachea are the most important hubs with the highest variety of Th subset regulator expression, suggesting their highest activities in these tissues. Two of the tissues such as muscle and trachea are our newly identified most active Th subset hubs in non-classical immune tissues.

### Chronic Metabolic Diseases and Autoimmune Diseases Increase Multiple Th Subset Regulators but Decrease Treg Regulators; and Familial Hypercholesterolemia and Lupus Do Not Increase Th1 Regulators

Previously, we reported that the presence of CD4+ CD25+ Treg in proatherogenic mouse model apolipoprotein E-deficient (ApoE−/−) mice were significantly decreased (13). Moreover, we also suggested that pathological conditions re-shape physiological Tregs into pathological Tregs (1, 19). Here, we hypothesized that in addition to Treg regulation, the expression of all the Th subset regulators are modulated differentially in chronic metabolic diseases and autoimmune diseases compared to healthy conditions.

To test this hypothesis, we examined the gene expression data extracted from microarray experiments deposited in the NIH-Geo Datasets. These microarrays were conducted on insulin-resistant obese patients’ omental and subcutaneous adipose tissues. The results in the Table S1 in Supplementary Material showed that in omental and subcutaneous adipose tissues, insulin resistance inhibits the expression of Treg (forkhead family of transcription factor O1, FoxO1) and Th22 (ligand-activated helix-loop-helix transcription factor and aryl hydrocarbon receptor, AHR) regulators but promotes the expression of other Th subset regulators in visceral adipose tissues. In addition, we found that type 2 diabetes (45) and hyperlipidemia promote multiple Th subset gene expression in mouse liver, adipose tissue, and spleen (Table S2 in Supplementary Material).

Peroxisome proliferator-activated receptor γ (PPARγ) agonist rosiglitazone treatment can predispose the heart to failure in human and rodent models. Our data reveals that rosiglitazone treatment in ApoE−/− mice significantly increased the expression of Th subset regulators, including signal transducer and activator of transcription 1 (Stat1) for Th1, c-Maf inducing protein (Cmip) for Th2, PU.1 is an ETS-domain transcription factor (Spi1) for Th9, transcription Factor Maf for Tfh, retinoid-related orphan receptor alpha (Rora) for Th17, FOXO1 and FOXP3 for Treg, and Ahr for Th22 (Table S3 in Supplementary Material). Furthermore, we found that hyperlipidemia promotes Th subset gene expression in ApoE−/− mouse aortic adventitia (Table S4 in Supplementary Material) and that aged ApoE−/− mice express fewer Th subset regulators (Table S5 in Supplementary Material). Finally, we found that in CD3+ T cells, familiar hypercholesterolemia upregulates all the Th subset genes but not Th1 (Table S6 in Supplementary Material). These findings are summarized in Table 7.

**Table 7:** Metabolic cardiovascular diseases increase multiple Th subset regulator expression but inhibit Treg regulator expression (Tables S1–S6 in Supplementary Material).

Disease	Tissue	Result
Obesity	Omental and subcutaneous adipose	Insulin resistance inhibits the expression of Treg and Th22 regulators but promotes the other Th subsets regulators in patients’ visceral adipose tissues

Type2 diabetes and hyperlipidemia	Liver, adipose tissues, and spleen	Type 2 diabetes and hyperlipidemia promote multiple Th subset gene expressions

Failing heart	Heart	Falling heart induced in apolipoprotein E-deficient (ApoE−/−) mice with PPARγ agonist rosiglitazone significantly increase the expressions of Th subset regulators

ApoE−/− mice	Aorta	Hyperlipidemia promotes Th subset gene expressions in aortic adventitia

	Blood	Hyperlipidemia in ApoE−/− blood increase a few Th subset genes in comparison to wild-type mouse blood

Familial hypercholesterolemia	CD3 T cell	Familial hypercholesterolemia upregulate all the Th subset genes except that of Th1

We also examined the Th subset regulator expression in two prototypic autoimmune diseases such as systemic lupus erythematosus and psoriasis. We found (Tables S7–S9 in Supplementary Material) that systemic lupus erythematosus increases Th2, Th9, Tfh subset genes but decrease Th1 and Treg genes in blood. Th1 cytokine interferon-γ therapy in patients with lupus increases Th1 and Th17 subset genes. We then found that in skin biopsy psoriasis increases all Th subset regulators except Treg genes, which were correlated with the recent report on increased Th17 and Th22 in patients with psoriasis (46). A summary of the findings are presented in Table 8.

**Table 8:** Autoimmunity diseases promote multiple Th subset regulators but inhibit Treg regulators (Tables S7–S9 in Supplementary Material).

Disease	Tissue	Result
Systemic lupus erythematosus	Blood	Increase Th2, Th9, and TFh subset genes but decrease Th1 and Treg genes
		Th1 cytokine IFN-υ therapy in lupus patients increases more Th1 and Th17 subsets than non-treatment control in blood cells

Psoriasis	Skin biopsy	Induces all the Th subset genes except Treg genes

Taken together, metabolic diseases and autoimmune diseases increase the expression of multiple Th subset regulators but decrease Treg regulator expression. Also, familial hypercholesterolemia and lupus do not increase Th1 regulator expression, which cannot exclude the possibility of local increase and increased function of Th1 without increased expression in familiar hypercholesterolemia and atherosclerosis (47).

### Severe Trauma Injury Increases Th2, Th9, Tfh, and Th17 Subset Regulators but Decreases Th1 and Treg Regulators in Blood; and Burn Injury Decreases All Th Subset Regulators Except Th22 Regulators

It has been reported that severe trauma injury (40) and burn injury (41) cause a genomic storm that lead to suppression of the genes involved in adaptive immunity. Thus, we hypothesized that the expression of Th subset regulators are modulated in severe trauma and burn injury. As summarized in Table 9, we found that severe trauma increases the expression of Th2, Th9, and Th17 subset regulators, but decreases the expression of Th1 and Treg regulators, suggesting that severe trauma decreases pro-inflammatory Th1 and anti-inflammatory/immunosuppressive Treg at the same time, which were correlated well with decreased presence of Th1 and Treg in smoke inhalation-induced lung injury (48). By comparison, we found that burn injury in patients decreases the expression of all the Th subset regulators except Th22 regulators (Table S11 in Supplementary Material). IL-22 is produced by a subset of human skin-homing memory T cells (49). Also, IL-22 is a master homeostatic cytokine preserving the integrity of boundary organs and tissues, especially for epithelial cell regeneration (50). Therefore, for the first time, our results have demonstrated that the expression of IL-22 generating Th22 (51) regulators sustain burn injury-induced heavy stress and may play a critical role for tissue recovery from burn injury.

**Table 9:** Similar to lupus patients, severe trauma increase Th2, Th9, TFH, and Th17 subset regulator expressions but decreases Th1 and Treg regulator expressions; but burn injury decreases all the Th subset regulators except Th22 regulators (Tables S10 and S11 in Supplementary Material).

Disease	Tissue	Result
Severe trauma		Increase Th2, Th9, TFH, and Th17 subsets but decreases Th1 and Treg

Burn injury	White blood cells	Burn Injury in patients decreases all the Th subsets except Th22

### Vascular Cells Are Functional As Innate Immune Cells, Express Numerous Th Subset-Promoting Cytokines and Stimulate Th Regulator Expression Other than Treg Regulator Expression

We recently proposed a new paradigm that endothelial cells are conditional innate immune cells, which actively participate in both innate and adaptive immune responses (52). To consolidate the new model, we hypothesized that vascular cells respond to cardiovascular disease risk factors and endogenous metabolites-derived danger signals and differentially modulate Th subset regulator expression compared to healthy condition. As summarized in Table 10, we found that aortic endothelial cells activated by proatherogenic stimuli including lipopolysaccharide (LPS) (8), oxidized low-density lipoprotein (oxLDL) (53), and oxidized 1-palmitoyl-2-arachidonoyl-sn-glycero-3-phosphocholine (oxPAPC) upregulate more effector Th subset regulators than Treg regulators (Table S12 in Supplementary Material). In addition, we found that vascular smooth muscle cells (VSMCs), in response to pro-inflammatory stimuli such as tumor necrosis factor-α (TNF-α), and a combination of TNF-α and lymphotoxin beta-receptor (α-LTβR) monoclonal antibody (54) upregulate more Th1, Th9, Tfh, Th17 regulators than Treg regulators (Table S13 in Supplementary Material). These results suggest that vascular cells including endothelial cells and VSMCs contribute more to non-Treg Th subset development than Treg development in pro-inflammatory conditions.

**Table 10:** As innate immune cells, vascular cells secrete numerous Th subsets-promoting cytokines and induce the expression of Th subset regulators except Treg regulators (Tables S12 and S13 in Supplementary Material).

Disease	Tissue	Result
Atherosclerosis	Aortic endothelial cells	Aortic endothelial cells activated by pre-atherogenic and stimulate to secrete Cytokines that promote more effector Th subsets than Treg

Atherosclerosis and myocardial infraction	Smooth muscle cells	Vascular smooth muscle cells in response to inflammatory stimulates, secrete cytokines that promote more Th1, Th9, TFH, Th17 than Treg

### GATA3 and HDAC6 Promote, but BCL6 Inhibits Th Subset Regulator Expression in Treg, and Regulate Treg Plasticity and Heterogeneity

It has been reported that Treg stability can be undermined or endorsed by different type 1 cytokines (55). Treg transcriptome is not stable, which contributes to Treg plasticity in gene transcription and immunosuppressive function (1). For example, atherosclerosis-driven Treg plasticity leads to formation of a dysfunctional subset of plastic Th1 cytokine interferon-γ (IFN- γ) Th1/Treg (26). In addition, IL-17A-secreting Treg was also identified (27). Moreover, myocardial infarction increases Treg but their function is compromised (28). Thus, we hypothesize that non-Treg Th subset-associated transcription factors not only regulate the development of non-Treg Th subsets but also functionally collaborate with Treg transcription factor FOXP3 in suppression of other Th subset regulator expression in Treg, and maintain Treg identity.

To test this hypothesis, we examined the expression of Th subset regulators in Treg in diseased conditions and in the presence of the mutations of various Treg-related regulators. As summarized in Table 11, we found that diabetes “switch” Treg to express other Th subset regulators, which is correlated with recent reports on plastic Treg in type 1 diabetes (56) and autoimmune arthritis (57). In addition, we also found that induced Treg are less likely than natural Treg to express non-Treg Th subset regulators except Th1 cytokine IFN- γ. Our results suggest that induced Treg have higher probability than natural Treg to become IFN- γ-secreting Treg rather than other Th subsets, which was not reported in the original paper (58). Moreover, deficiency of Treg in Foxp3 mutant Scurfy mice promotes the expression of other Th subset regulators, especially Th1 transcription factors such as Tbx21, transcription factor Eomesodermin (Eomes) (59), and Th22 regulator Ahr. This suggests that Foxp3 is functional in suppressing the expression of Th1 and Th22 subset regulators in Treg; and Foxp3-deficient Treg do not revert into conventional effector CD4+ T cells but constitute a unique cell subset (60).

**Table 11:** Several master regulators, including Foxp3, Xbp1, GATA1, CTLA-4, GATA3, BCL6, HDAC6, and PPARγ, modulate Th subset regulator expressions in Treg and other Th subsets.

Conditions	Tissue	Result
Plasticity of Treg	Spleen	Diabetes “switch” Treg to express other Th subset genes
		Induced Treg are less likely to express Th subset genes except IFNγ relative to natural Treg. This suggest that induced Treg shows less plasticity than natural Treg. Induced Treg have higher potential than natural Treg to become IFNγ secreting Treg

Scurfy mutation Foxp3−/−	Lymph node cells	Deficiency of Treg in Scurfy mice promotes the development of other TH subsets in CD4+ T cell populations

	T cell	CD4+ T cells in Foxo3 scurfy mice require IL-2 to express Th subset genes
		MicroRNA processing enzyme Dicer inhibits other Th subset genes expression

Xbp1 knockout	Spleen	Xbp1 inhibits Treg and expresses other Th subset genes
Gata1 knockout		GATA1 inhibits Treg to express other Th subset genes

CTLA-4 knockout	T cell	CTLA-4 inhibits Th subset gene expression but promotes IL-2 and other Treg gene expression

Gata3 knockout	Treg	Gata3 promotes Th subset gene expression in Treg and Treg plasticity
Bcl6 and HDAC6		Bcl6 and HDAC6 inhibits Th subset gene expression in Treg and Treg plasticity

Treg-Pparγ. Mut	Lymph nodes	Treg-specific PPARγ INHIBITS Th subset gene expression in adipose tissue and lymph node
	Visceral adipose tissue	

Multiple factors “lock in” the transcriptional signature of Treg (61). Along the same line, we found that X-box binding protein 1 (Xbp1, a bZIP domain transcription factor) (61), Gata-binding factor 1 (Gata1, a transcription factor) (61), microRNA processing enzyme Dicer (62), cytotoxic T-lymphocyte-associated protein 4 (Ctla4) (63), and peroxisome proliferator-activated receptor gamma (PPARγ) (64) can also inhibit the expression of other Th subset regulators in Treg, and maintain Treg identity. This observation is similar to the previous report that showed impaired presence of Treg in PPARα KO mice (65).

Finally, we found that Th2-associated transcription factor Gata3 globally promotes the expression of Th1-associated transcription factors Tbx21, Th17-associated TF Rorc, Treg-transcription factors FoxO3 and FoxO1, and Tfh-associated TF BCL6in Treg (Table S24 in Supplementary Material). Of note, functional counteraction of Foxp3 and Gata3 was reported previously (66), but our findings of Gata3 promoting the expression of Tbx21, Rorc, and BCL6 are novel.

Our previous paper reported that Treg have high expression of epigenetic master gene histone deacetylase 6 (HDAC6) than other T cells (19, 20). However, our results here showed that HDAC6 promotes the expression of Th1-associated TF Tbx21, Th17 associated TF Rora and Th2 inducing cytokine IL-4 in Treg. These results are well correlated with the previous report indicating that inhibition/deficiency of HDAC6 improve FOXP3+ Treg function (67). Taken together, our findings suggest that in normal conditions, HDAC6 promotes Treg plasticity and Treg heterogeneity.

We also found that Tfh-associated transcription factor Bcl6 deficiency upregulates Th1-associated TF Tbx21, Th2-associated TF GATA3, Tfh-associated TFs Batf, and Maf, Th17-associated TF Rora, Treg-associated TF FOXP3, and Th22-associated TF Ahr in Treg, suggesting that BCL6 globally suppresses Treg plasticity. Of note, BCL6 mediated inhibition of Th2 inflammatory activity of Treg was reported previously (68), but the rest of our findings are novel.

**Figure 5:**
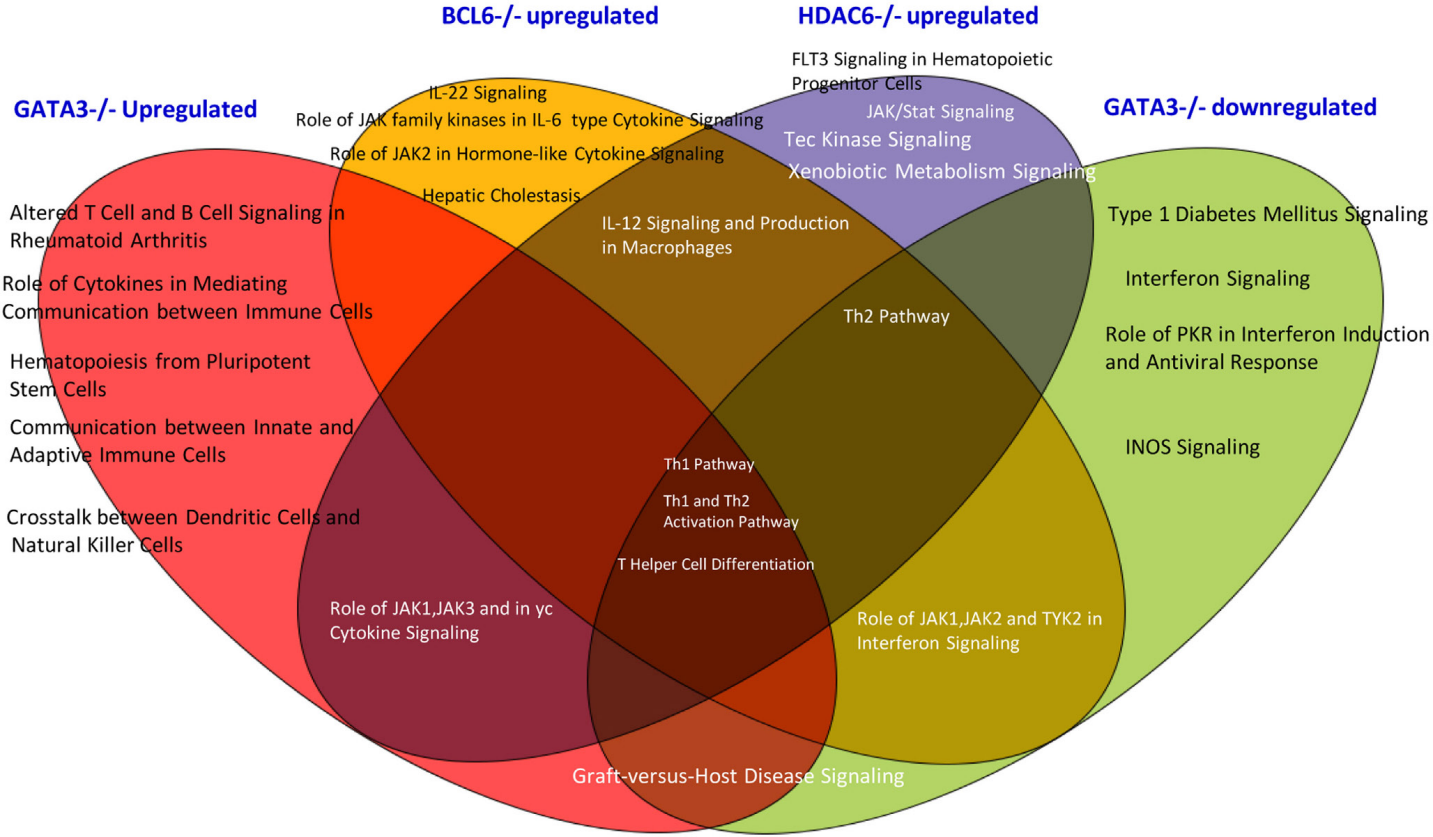
The Venn diagram analysis. The Venn diagram shows that Treg plasticity regulators Gata3, Blc6, and Hdac6 shre Th1 pathway and Th differentiation pathways.

In order to find out which pathways are involved in Gata3, Bcl6, and HDAC6 regulation of Treg plasticity, we used the Ingenuity Pathway Analysis to determine the pathways involved. As shown in Figure 5, we found that among three gene deficiencies-induced pathways in Treg, all the three pathways regulate Th1 polarization, Th1 and Th2 activation processes, and T helper cell differentiation. Each of three master gene mutations have four to five specific pathways and have five to six pathways shared with other master genes regulated pathways. These results suggest that: first, GATA3 is not only responsible for developing Th2; BCL6 is not only responsible for developing Tfh subset, GATA3 also promotes Treg plasticity; second, in contrast to GATA3, BCL6 collaborates well with Treg-specific TF FOXP3 in suppression of other Th subset regulator expression in Treg; and third, HDAC6-mediated removal of histone acetylation, presumably at specific amino acid residue(s) in histones, promotes Treg plasticity and Treg heterogeneity.

### Gata3 and Bcl6 Inhibit the Expression of MHC Class II, and T Cell Co-Signaling Receptors in Treg and “Trans-Differentiate” Treg into Antigen-Presenting Cell (APC)-Like Treg

Recent reports showed that an increased number of cell types that are capable of acting as atypical antigen-presenting cells (APC) and present antigens to major histocompatibility complex (MHC) class II molecules to CD4 + T cells (69). CD4 + T cells are among atypical APCs. However, it is unknown whether plastic Treg can serve as atypical APC. We hypothesize that plastic Treg not only change into pro-inflammatory cytokine IFN-γ-secreting Th1-like Treg but also “trans-differentiate” into atypical APC. It has been well accepted that MHC class II molecules present MHC II-restricted antigen epitopes to bind to T cell antigen receptor (TCR) to deliver the signal 1 for T cell activation (10). We examined whether MHC class II molecules are upregulated in plastic Treg. As shown in Table 12A, among 14 MHC class II molecules we found in the NIH-NCBI-Gene database, seven MHC class II molecules are upregulated in Gata3 KO Treg. In addition, we also found that five MHC class II molecules are upregulated in Bcl6 KO Treg; and five MHC class II molecules are downregulated in Hdac6 KO Treg, suggesting that Gata3 and Bcl6 inhibit MHC class II molecule expression in Treg, but HDAC6 promotes the expression of MHC class II molecules in Treg.

**Table 12:** The mutations of three master regulators such as GATA3, BCL6 and HDAC6 modulate the expression of MHC class II molecules (signal 1 for T cell activation) and co-stimulation/co-inhibition receptors (signal 2 for T cell activation) in Treg.

(A) Treg plasticity regulators GATA3−/−, BCL6−/− upregulate, and HDAC6−/− downregulate the expression of mouse major histocompatibility complex (MHC) genes in the Treg, suggesting that the plastic Treg become antigen-presenting cell (APC)-like Treg to deliver the signal #1 to activate T effector cells				
Official symbol	Gene ID	GSE39864	GSE40493	GSE27896
		
		GATA3	BCL6	HDAC6
Cd74	16149	1.33	5.16	
Ciita	12265			−1.24
H2-Aa	14960	1.45	1.99	−1.28
H2-Abl	14961	1.42	2.39	
H2-DMa	14998	1.45		−1.38
H2-DMb1	14999			
H2-DMb2	15000	1.22		−1.34
H2-Ea-ps	100504404			
H2-Eb1	14969	1.41	1.47	−1.23
H2-Eb2	381091			
H2-K1	14972		1.6	
H2-Oa	15001	1.48		
H2-Ob	15002		−1.66	
Mrl	15064

*GSE 39864 (Gata3 KO vs WT T-reg), GSE 40493 (Bcl6 KO vs WT T-reg), GSE 27896 (Hdac6 KO vs WT T-reg)*.				

**(B) Treg can convert to antigen-presenting cell (APC)-like Treg and upregulate APC-expressed T cell co-stimulation/co-inhibition receptors to deliver the signal 2 to activate T effector cells**				

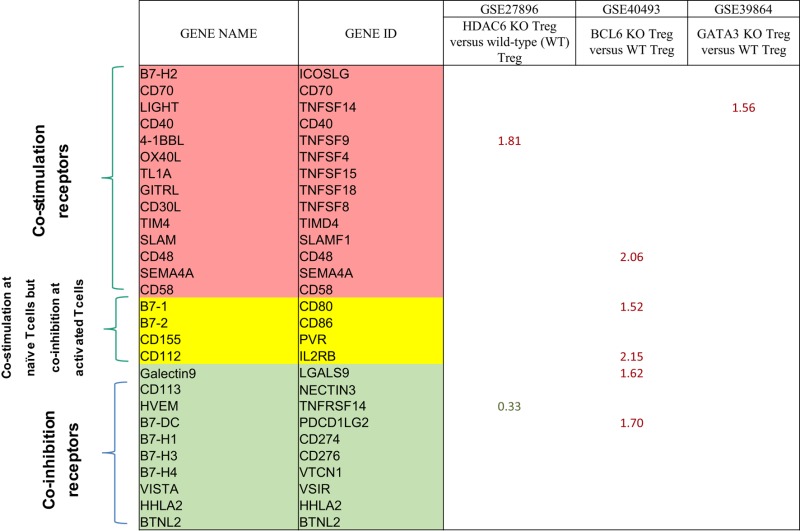				

**(C) Treg with increased plasticity can act as “antigen-presenting cell (APC)-like Treg” by upregulating co-stimulation co-receptors such as LIGHT, 4-1BBL, CD48, B7-1, and CD112, and downregulating co-inhibition co-receptor HVEM**				

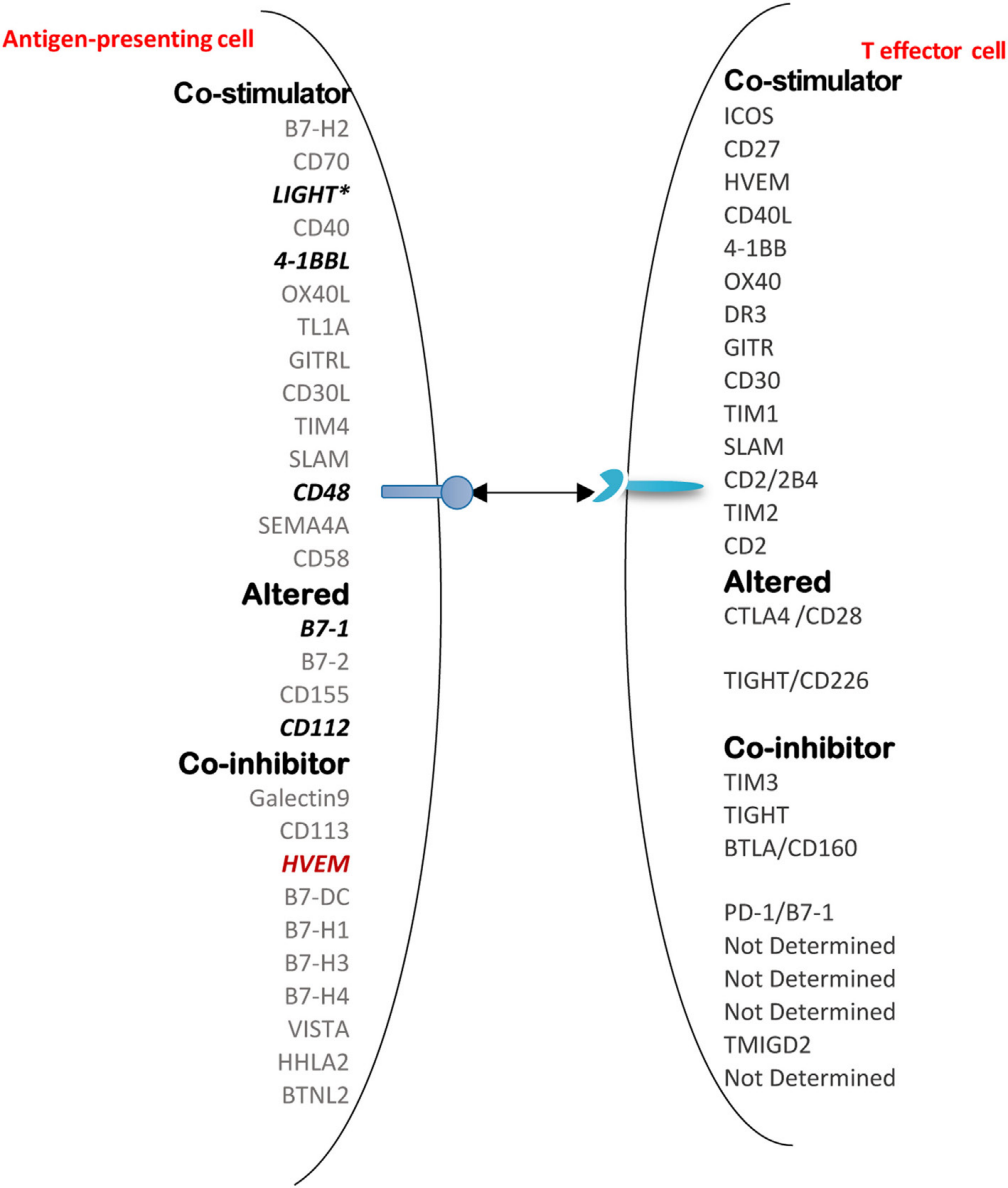				

*The co-signaling receptors shown in bold letters were upregulated in T-reg with increased plasticity*.

Moreover, it has been well-characterized that T cell co-signaling receptors including co-stimulation receptors and co-inhibition receptors bind to the intrinsic co-receptors on the effector T cell surface to trigger the signal 2 pathways for T cell activation (70–72). Thus, we examined whether the expression of T cell co-signaling receptors are upregulated in Gata3−/− Treg, Bcl6−/− Treg, and/or Hdac6−/− Treg. As shown in Table 12B, we found that: *first*, among 28 co-signaling receptors expressed on the cell surface of classical APCs (70–72), co-stimulation receptor 4-1BBL is upregulated in Hdac6 KO Treg; *second*, co-stimulation receptor LIGHT is upregulated in Gata3 KO Treg; *third*, co-stimulation receptors CD48, B7-1 (CD80), CD112, co-inhibition receptors galectin 9 and B7-DC are upregulated in Bcl6 KO Treg; and *fourth*, one co-inhibition receptor HVEM is downregulated in Hdac6 KO Treg, suggesting that Hdac6, Bcl6 and Gata3 inhibit the expression of co-signaling receptors in Treg (Table 12C). Of note, these results showed that co-stimulation receptor 4-1BBL is upregulated but five MHC class II molecules are downregulated in the Hdac6 KO Treg, suggesting that HDAC6 is not potent in regulating the Treg changes to atypical APC. Taken together, our results showed that (1) Gata3 and Bcl6 collaborate in inhibiting Treg plasticity and conversion into atypical APC; (2) mutations of Gata3 and Bcl6 “trans-differentiate” Treg into atypical APC; (3) Bcl6 inhibit Treg plasticity into Th1-like Treg and atypical APC but Hdac6 only promotes Treg plasticity into Th1-like Treg; and (4) GATA3 determine the fate of plastic Tregby controlling whether it will convert in to either Th1-Tregor APC-Treg (Figure S2 in Supplementary Material).

## Discussion

In last 30 years, significant progress has been made in understanding of CD4 + T cells. Naïve CD4 + T cells can be differentiated/polarized into at least nine or 10 terminally differentiated subsets including Th1, Th2, Th9, Tfh (21), Th17, Treg, Th22 (1, 22), Th25 (23), CD4 + cytotoxic T cells (CD4 + CTL) (24) as well as innate lymphoid cell subsets (73). However, several important questions remain to be addressed including: (a) the tissue expression profile of Th subset regulators in human tissues; (b) how Th subset regulators are globally changed in various diseases; and (c) in addition to converting into Th1 and Th17 that have been reported, whether Treg plasticity can enhance T cell immune responses by acting as antigen-presenting cells (APC-Treg).

To address these issues, we took an experimental data mining approach that we pioneered (32, 74, 75) and examined the panoramic tissue expression patterns of as many as 61 Th subset regulators in physiological and pathological conditions. We made the following findings: (1) All the Th subset regulators are differentially expressed in various human tissues including heart and vascular tissues at physiological conditions; (2) Chronic metabolic diseases and autoimmune diseases increase multiple Th subset regulators but decrease Treg regulators. Familial hypercholesterolemia and lupus do not increase Th1 regulators; (3) Severe trauma increases Th2, Th9, Tfh, and Th17 subset regulators but decreases Th1 and Treg regulators in blood. In contrast, burn injury decreases all Th subset regulators except Th22 regulators; (4) Vascular cells function as innate immune cells [as we reported previously for endothelial cells (52)], therefore, express numerous Th subsets-promoting cytokines and promote Th regulator expression other than Treg regulator expression; (5) GATA3 and Hdac6 promote, but Bcl6 inhibits, Th1 subset regulator expression in Treg by regulating Treg plasticity and heterogeneity; and (6) Furthermore, our results indicated that HDAC6 promotes but GATA3 and Bcl6 inhibit the expression of MHC class II molecules T cell co-signaling receptors expression in T-reg and “trans-differentiate” Treg into APC-like Treg. Therefore, based on our findings, we conclude that increased HDAC6 activity and reduced Bcl6 expression regulate the plasticity and heterogeneity of T-reg, but Gata3 expression level is the main determinant of Tregdifferentiation in to either Th1-Treg or APC-Treg.

It has been a common practice to perform microarray analysis and look for large fold changes (>2-folds) in gene expression with the treatment of interest. However, a recent report showed that in searching for the mechanism underlying NZW mouse Treg instability, gene expression profiles highlight specific differences in the transcriptome of NZW Tregs compared with those of other strains, but no single defect could obviously account for the Foxp3 instability. Rather, NZW Tregs show a general upregulation of transcripts normally repressed in Treg cells, which suggest that this network-level bias may account for NZW Treg instability (76). Along the same line, an innovative concept was presented and claimed that gene regulatory networks are sufficiently interconnected as such that all genes expressed in disease-relevant cells are liable to affect the functions of core disease-related genes. In addition, it was stated that most heritability can be explained by effects on genes outside the core pathways. Our findings support this new concept in the following ways: low levels of expression changes (<2.0-folds) of a group of functional related master genes, especially transcription factors such as Treg-associated FOPX3, can be functionally significant if the *p* values of gene expression are statistically significant (< 0.05). FOXP3 is a transcription factor in determining the biogenesis and immunosuppressive function of Tregs (1, 3). Deficiency of FOXP3 leads to failed development of Tregs (1); and the levels of FOXP3 in Tregs reflect their functional status (77).

We used an experimental database mining approach that was pioneered and developed in our laboratory throughout the years (7, 32, 75, 78). By analyzing sequencing data from tissue cDNA libraries, we were able to study expression profiles of Th subset regulators in various tissues. Since this data is collected from cDNA cloning and DNA sequencing experiments rather than theoretical data derived from computer modeling, the findings presented in this paper are more relevant to the biological systems. Since the gene expressed sequence tag (EST) data deposited in the NIH-NCBI-UniGene database have been established based on DNA sequencing data, the data obtained by EST database mining are more precise in providing the tissue expression profiles of genes than traditional hybridization- and primer annealing-based approaches like Northern blots and RT-PCRs (32). Of note, since the UniGene database does not have many non-tumor cell line-related gene expression data in various gene deficiencies and stimulation conditions, we analyzed microarray-based gene expression data deposited in NIH-GEO Datasets to determine Th subset regulator expression changes under pathological conditions. Nevertheless, we acknowledge that further experiments such as qPCR, flowcytometry, immunoblots etc. are required to verify the tissue expression profile patterns of Th subset regulators and microarray results we report here.

**Figure 6:**
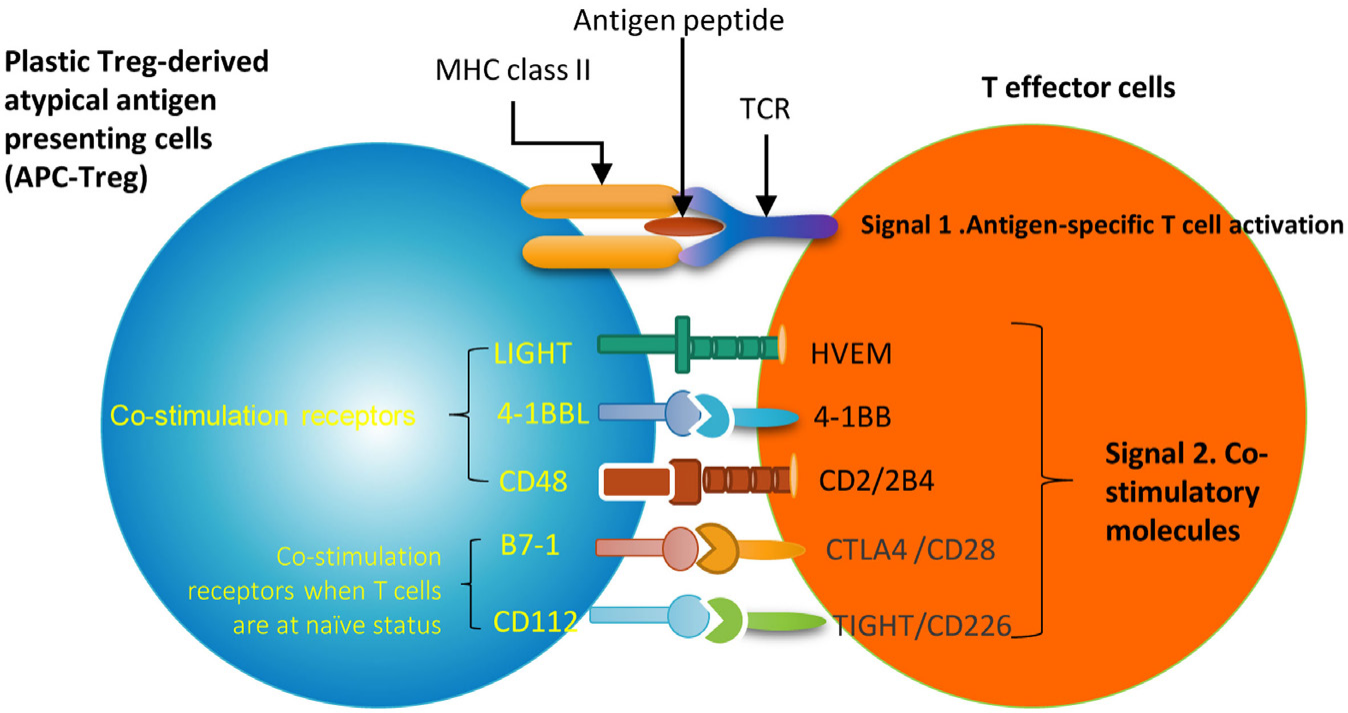
A novel hypothesis. Functional interaction between Treg-specific transcription factor Foxp3 and other transcription factors and/or master genes such as Gata3, Bcl6 and Hdac6 to maintain Treg identity and prevent Treg from losing the immune-suppressing activity and the conversion to APC-like Treg. Bcl6 deficiency upregulate expression of MHC class II molecules and T cell co-stimulators in Treg. This transformation of Treg to APC-Treg deliver T cell activation signal 1 *via* MHC class II/antigen epitope complex and T cell activation signal 2 *via* co-stimulation receptors LIGHT, 4-1BBL, CD48, B7-1 (CD80) and CD112. This may potentially promote inflammation and immune responses.

**Figure 7:**
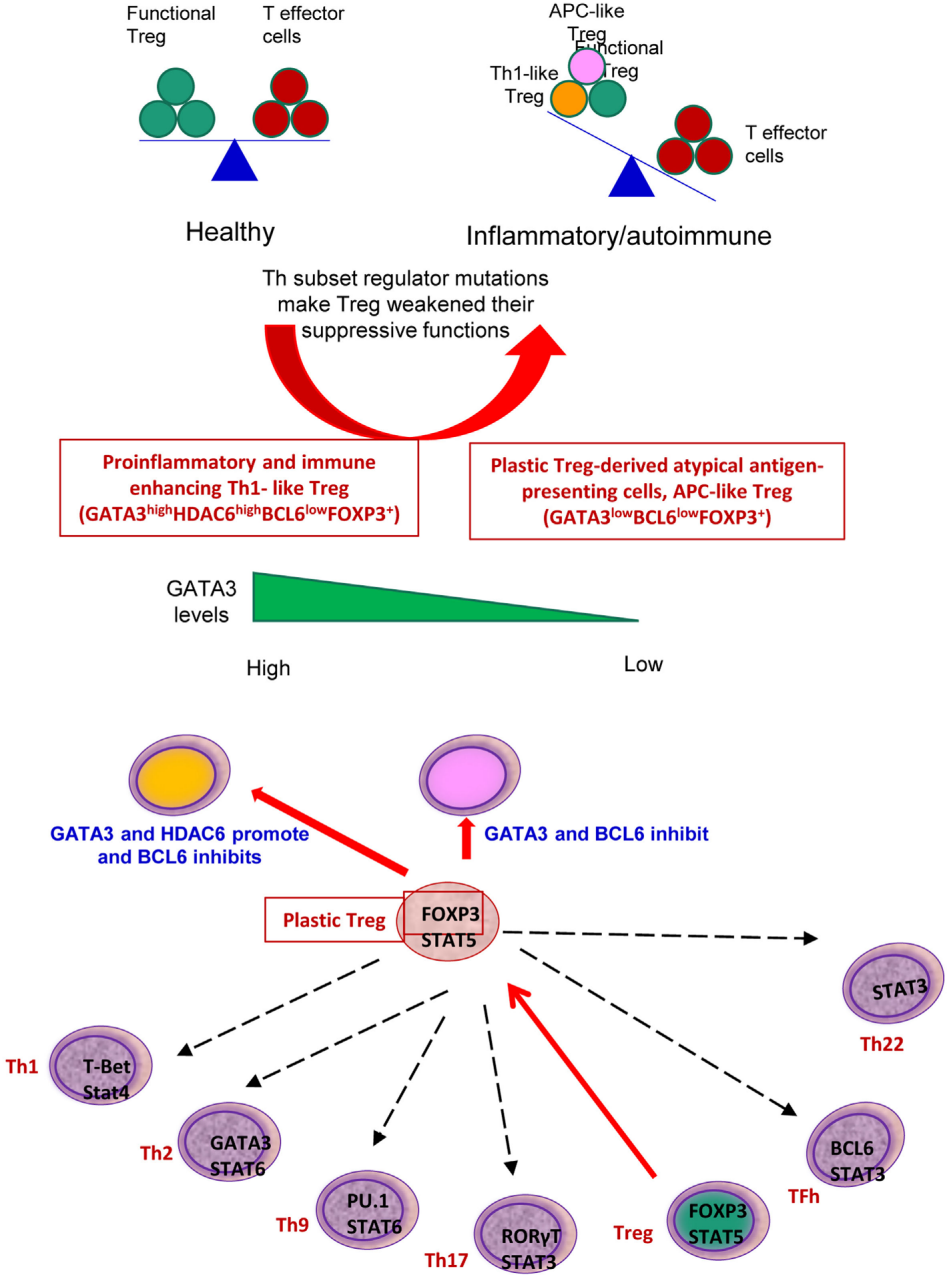
Newly proposed Treg stability/plasticity model: In order to maintain Treg stability, Treg need to express high level of FOXP3, HDAC6 and low levels of BCL6. GATA3 levels determine whether plastic T-reg will diverge in to Th1-Treg or APC-Treg lineage. When GATA3 expression is diminished in presence of high HDAC6 and low BCL6 levels, plastic T-reg tend to covert in to APC-Treg. However, when GATA3 expression is increased with high levels of HDAC6 and low Bcl6, T-reg convert in to Th1-Treg. Increased plasticity of T-reg weakens its immunosuppressive function and may facilitate inflammation and autoimmune reactions.

Recent progress demonstrated that in addition to inhibition of adaptive immune response, Treg also play a critical role in controlling various innate immune responses involved in cancers (2), inflammatory diseases including cardiovascular diseases and atherosclerosis (3), facilitate blood flow recovery after ischemia (4), control adipose tissue inflammation, and promote muscle repair (5). Herein, we report significant innovative findings, which allow us to propose a new working model (Figure 6): *first*, not only Treg have non-immune activities but also other Th subset may have this type of non-immune activities in various human tissues. Th subset regulators are not only expressed in secondary lymphoid tissues but also expressed in most other tissues, which may modulate Th subset tissue functions in a complex manner; *second*, as shown in our new Treg stability/plasticity model in Figure 7, Treg plasticity is not only toward Th1-Treg (26) and Th17-Treg (27) with the potential markers of GATA3^high^HDAC6^high^BCL6^low^FOXP3^+^, but also Treg plasticity upregulate MHC class II molecules and T cell co-stimulation receptors to function as antigen-presenting cells (APC-Treg with the potential markers of GATA3^low^HDAC6^high^BCL6^low^FOXP3^+^). Therefore, as mentioned above, our results demonstrated that while HDAC6 and Bcl6 are crucial to regulate Tregplasticity, Gata3 level in Tregdetermines whether the Tregis going to differentiate in to either Th1-Treg or APC-Tregin the presence of increased HDAC and low Bcl6 expression. Although atypical APC activity was reported previously (69), the deficiencies of GATA3 and BCL6 in Treg leading to upregulation of MHC class II molecules and T cell co-stimulation receptors has not been reported before. This suggests that BCL6 and specifically GATA3 inhibit Treg converting itself in to atypical APC. Of note, previous reports showed that FOXP3 can be presented by MHC class I and activate CD8 + anti-Treg (29, 30); and Treg-associated transcription factor FOXO3-upregulated tolerogenic dendritic cells express MHC class II and co-stimulation receptors (29, 30). Additionally, it was previously reported that expression of Bcl6 and IRF4 are required to specifically reduce subset-specific regulation of Th1 and Th17 cells, respectively. This report substantiates our findings that stability and maintenance of Bcl6 can be crucial to maintain Tregplasticity (79). Additionally, histone modifications were shown to regulate the Tregsignature as well. It was shown that decreased abundance of H3K4Me downregulated the Tregsignature genes while increased abundance of methylation at this specific site was observed in Th2-associated genes (80). To the best of knowledge, ours is the first report that claim acetylation of histone may also play a role in regulating the plasticity of T-reg. Our results have demonstrated for the first time that FOXP3 + Treg show plasticity and can convert into APC-Treg depending on the GATA3 expression level, therefore GATA3 is an important determinant of the fate of plastic Treg (Figure 7); *third*, a previous report showed that inducible Treg and Th17 cells, are more plastic than previously appreciated (81). However, our new findings demonstrated that inducible Treg express higher levels of Th1 cytokine IFNγ than natural Treg, suggesting that they have higher probability than natural Treg to be converted to Th1-Treg; and *fourth*, burn injury downregulates all the Th subset except Th22, indicating that Th22 is essential for tissue regeneration and repair. As we pointed out previously, data mining papers enable to generate new hypotheses and future experimental work is needed to consolidate our new findings. Nevertheless, our findings provide novel insights into Th subsets and Treg, which will eventually lead to future development of novel therapeutics for the treatment of inflammation, cancers, transplantation, autoimmune diseases and tissue regeneration.

## Author Contributions

KX carried out the data gathering, data analysis, figures/tables preparation, and manuscript writing. Other authors provided material input and helped revising the manuscript. XY supervised the experimental design, data analysis. All authors read and approved the final manuscript.

## Conflict of Interest Statement

The authors declare that the research was conducted in absence of any commercial or financial relationships that could be construed as a potential conflict of interest.

## Supplementary Material

Figure S1 Th subset regulator mRNA transcripts are differentially expressed in healthy human tissues.

Figure S2 Tfh transcription factor (TF) Bcl6 and Th2-TF Gata3 inhibit, but Hdac6 promotes Treg to become APC-like Treg. We conducted an Ingenuity Pathway Analysis to determine the signaling pathways that are regulated by the genes that are modulated by theses TF. The Venn diagram analysis showed that the majority of signaling pathways that are modulated by genes that are upregulated due to Gata3 deficiency and by the genes that are downregulated by Hdac6 deficiency are shared.
