# Supplementary material for: GATA3, HDAC6, and BCL6 Regulate FOXP3+ Treg Plasticity and Determine Treg Conversion into Either Novel Antigen-Presenting Cell-Like Treg or Th1-Treg
Source: Front Immunol. 2018 Jan 26;9:45. doi: 10.3389/fimmu.2018.00045 (PMC5790774; doi:10.3389/fimmu.2018.00045)

Supplemental data:.

Supplemental figure 1. Th subset regulator mRNA transcripts are differentially expressed in healthy human tissues.

Transcription factor:

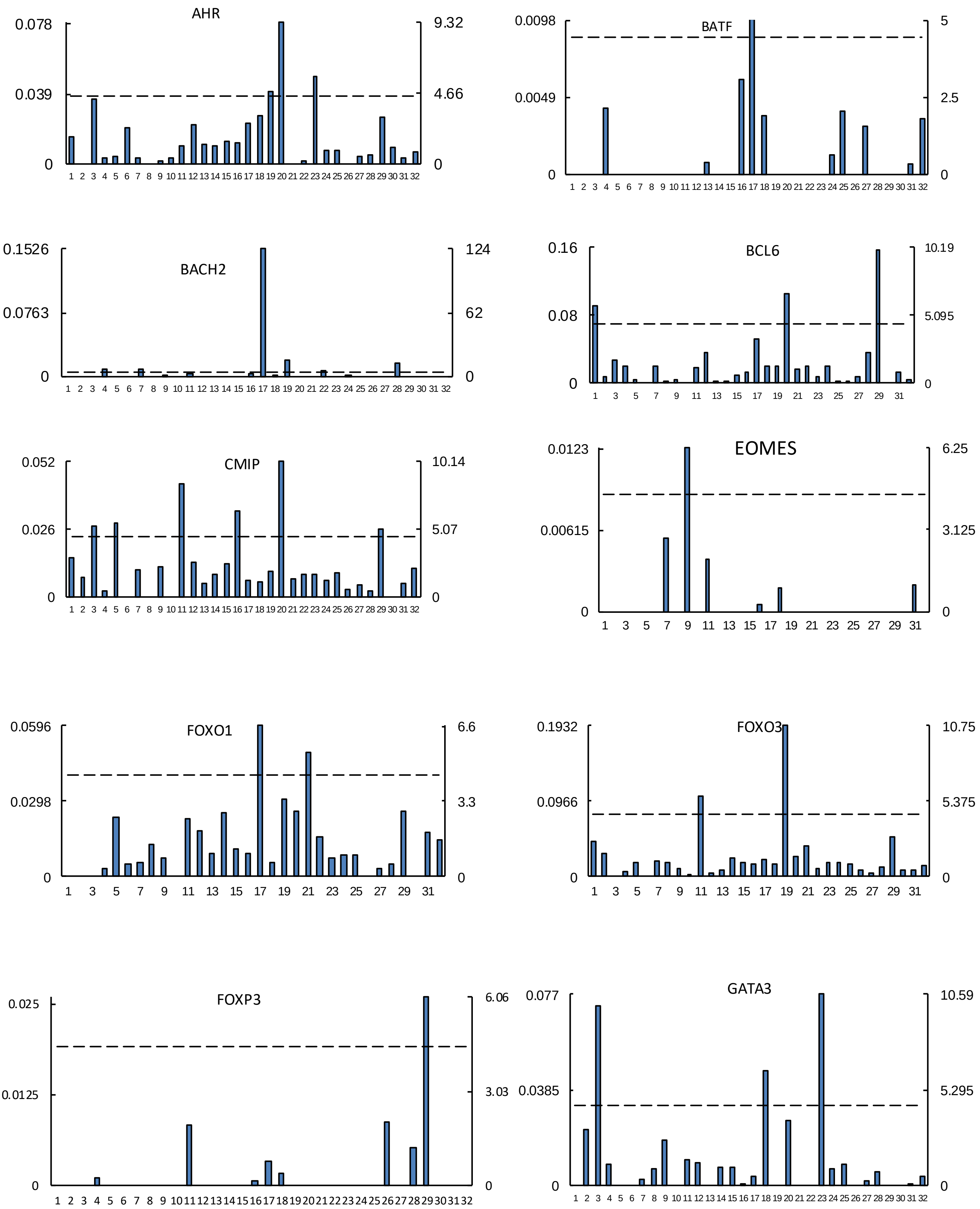

Continued:

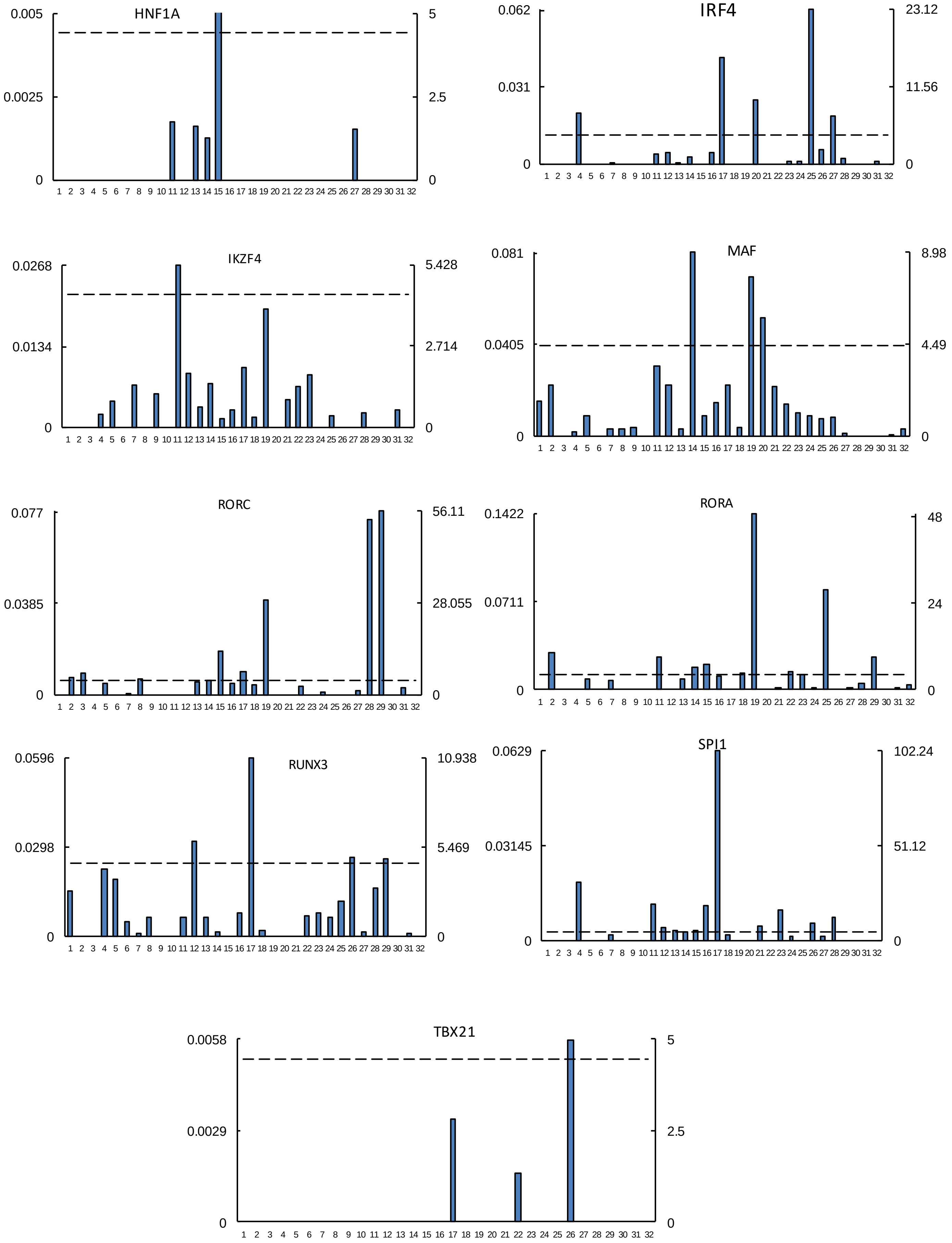
HNF1A
0.005
0.0025
0
5
2.5
0
IRF4
0.062
0.031
0
23.12
11.56
0
IKZF4
0.0268
0.0134
0
5.428
2.714
0
MAF
0.081
0.0405
0
8.98
4.49
0
RORC
0.077
0.0385
0
56.11
28.055
0
RORA
0.1422
0.0711
0
48
24
0
RUNX3
0.0596
0.0298
0
10.938
5.469
0
SPI1
0.0629
0.03145
0
102.24
51.12
0
TBX21
0.0058
0.0029
0
5
2.5
0
1 2 3 4 5 6 7 8 9 10 11 12 13 14 15 16 17 18 19 20 21 22 23 24 25 26 27 28 29 30 31 32

Transcription Activator:

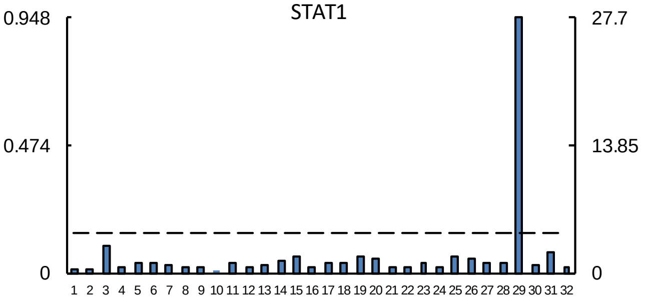

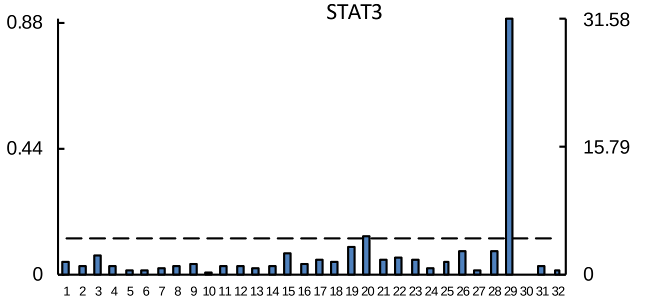

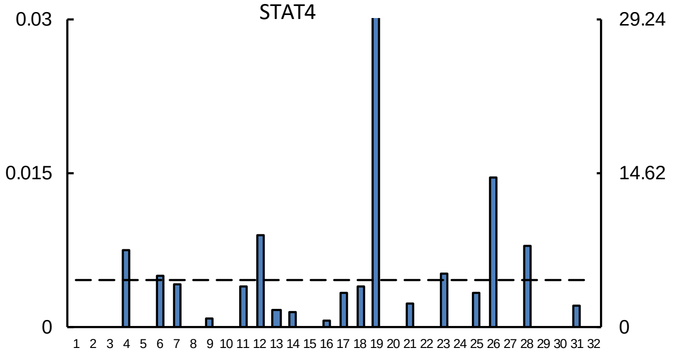

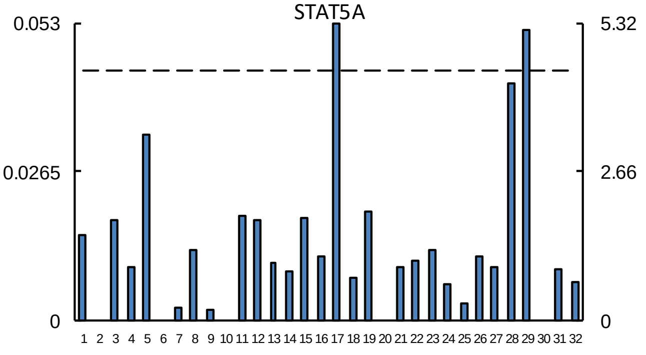

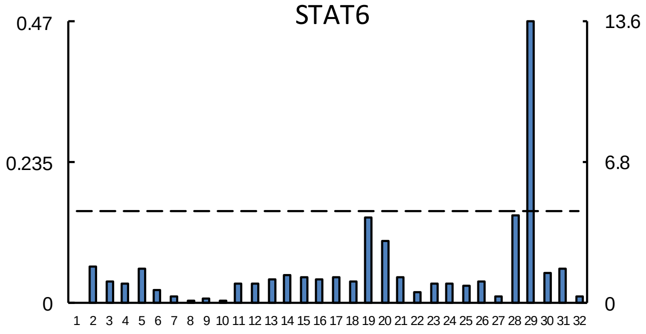

Transcription repressor:

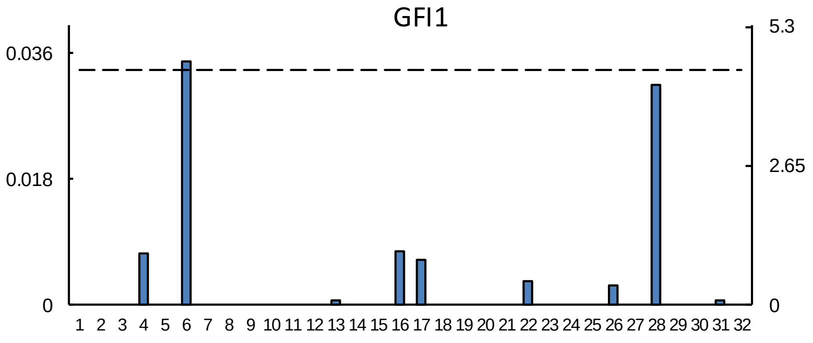

Signal transducers and transcription modulators:

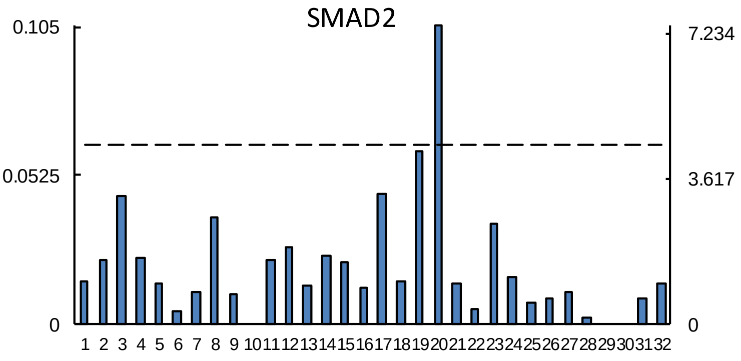

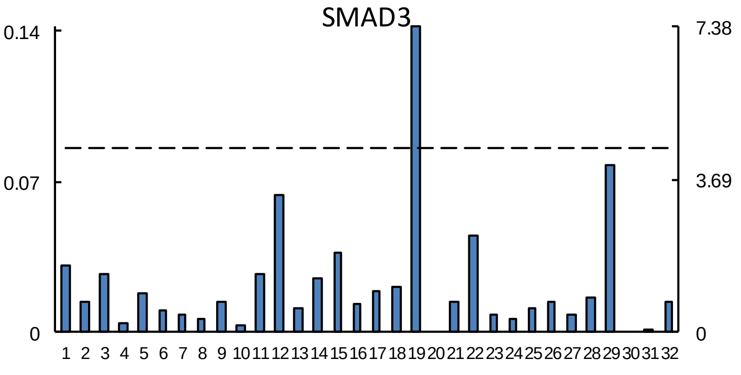

Cytokine:

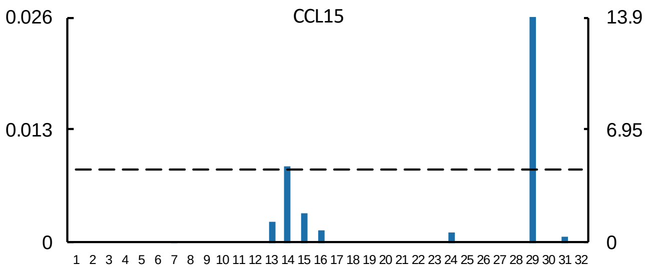
CCL15
0.026
0.013
0
13.9
6.95
0
1 2 3 4 5 6 7 8 9 10 11 12 13 14 15 16 17 18 19 20 21 22 23 24 25 26 27 28 29 30 31 32

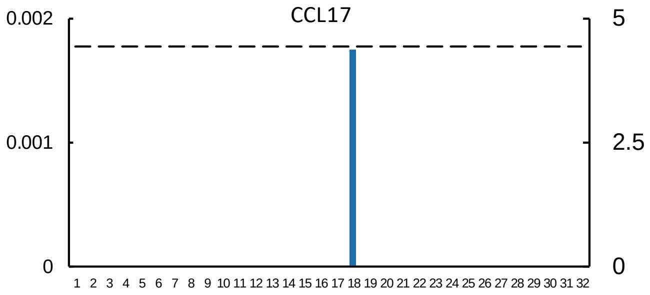
CCL17
0.002
0.001
0
5
2.5
0
1 2 3 4 5 6 7 8 9 10 11 12 13 14 15 16 17 18 19 20 21 22 23 24 25 26 27 28 29 30 31 32

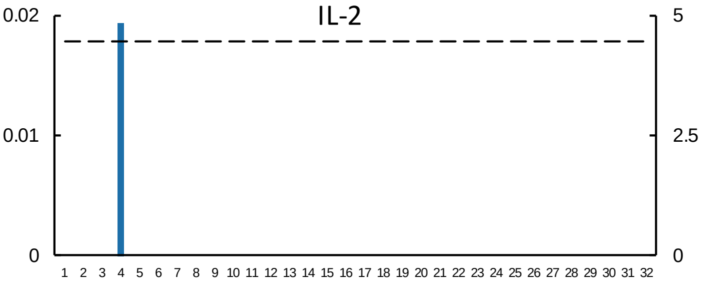
IL-2
0.02
0.01
0
5
2.5
0
1 2 3 4 5 6 7 8 9 10 11 12 13 14 15 16 17 18 19 20 21 22 23 24 25 26 27 28 29 30 31 32

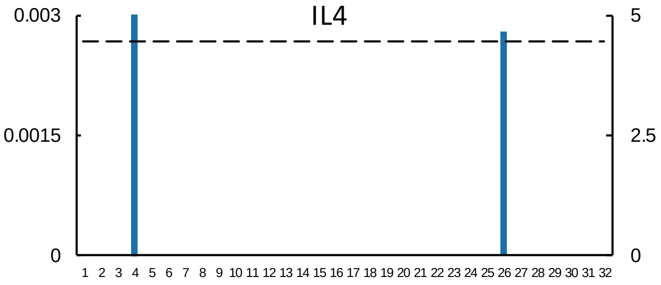
IL4
0.003
0.0015
0
5
2.5
0
1 2 3 4 5 6 7 8 9 10 11 12 13 14 15 16 17 18 19 20 21 22 23 24 25 26 27 28 29 30 31 32

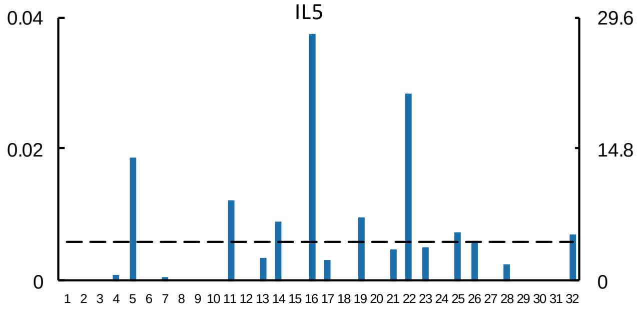
IL5
0.04
0.02
0
29.6
14.8
0
1 2 3 4 5 6 7 8 9 10 11 12 13 14 15 16 17 18 19 20 21 22 23 24 25 26 27 28 29 30 31 32

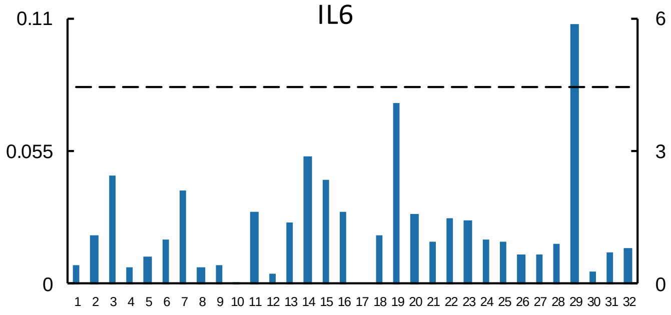
IL6
0.11
0.055
0
6
3
0
1 2 3 4 5 6 7 8 9 10 11 12 13 14 15 16 17 18 19 20 21 22 23 24 25 26 27 28 29 30 31 32

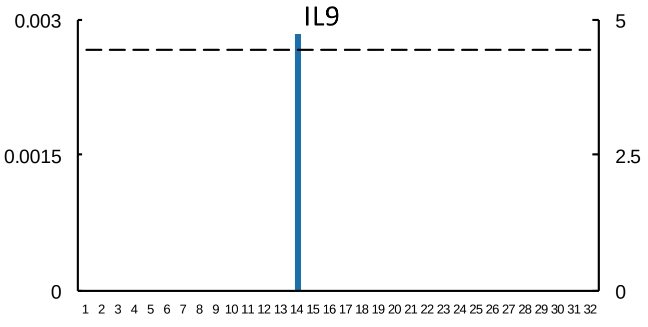
IL9
0.003
0.0015
0
5
2.5
0
1 2 3 4 5 6 7 8 9 10 11 12 13 14 15 16 17 18 19 20 21 22 23 24 25 26 27 28 29 30 31 32

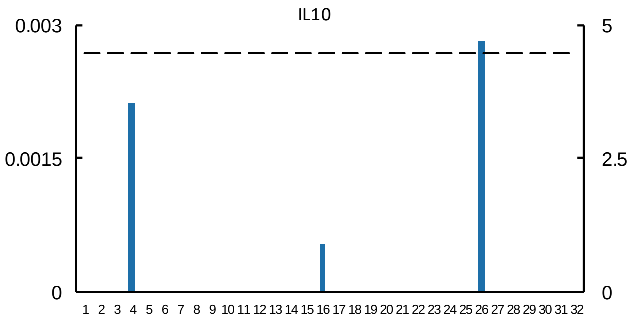
IL10
0.003
0.0015
0
5
2.5
0
1 2 3 4 5 6 7 8 9 10 11 12 13 14 15 16 17 18 19 20 21 22 23 24 25 26 27 28 29 30 31 32

Continued:

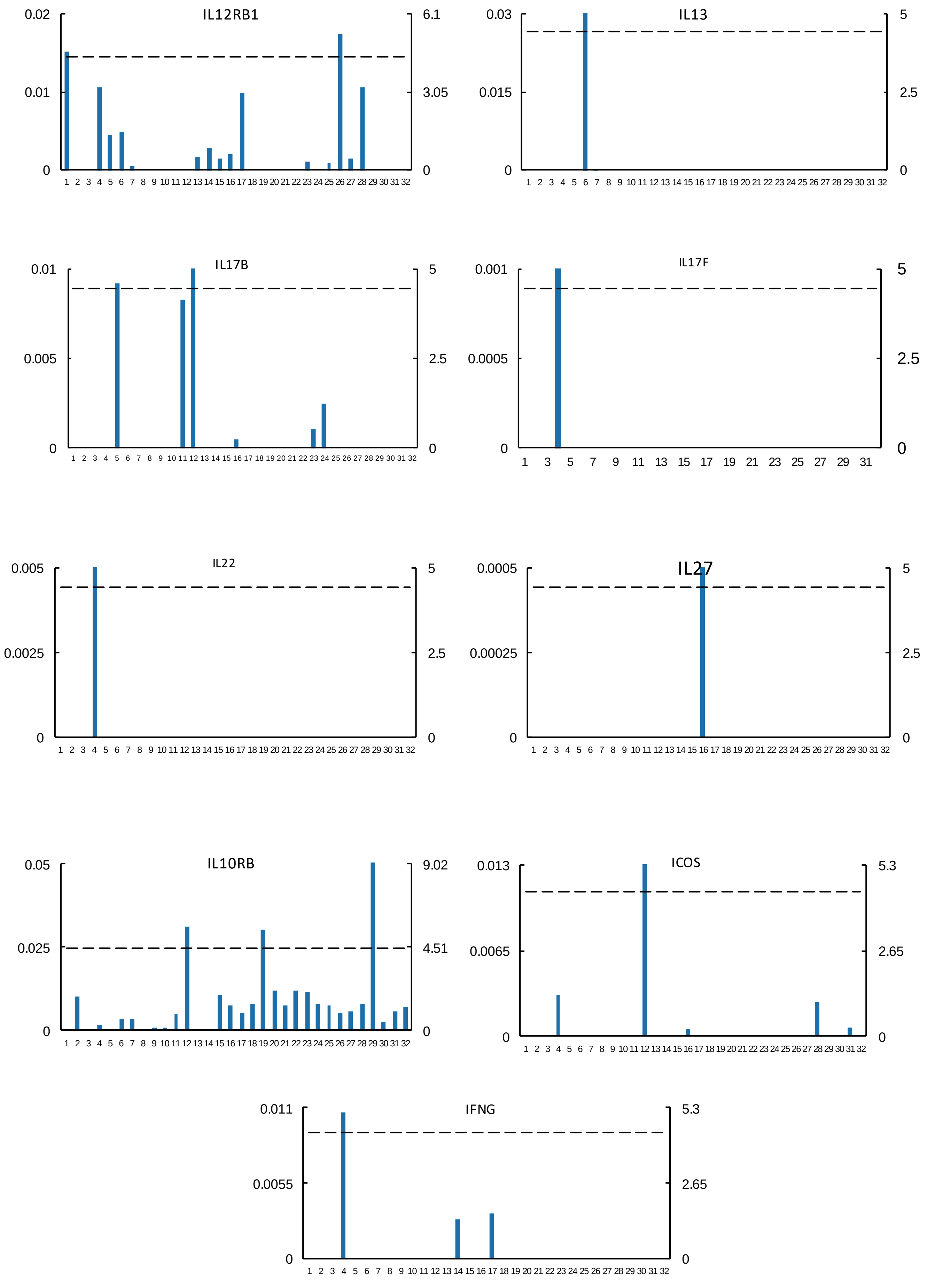
IL12RB1
0.02
0.01
0
6.1
3.05
0
IL13
0.03
0.015
0
5
2.5
0
IL17B
0.01
0.005
0
5
2.5
0
IL17F
0.001
0.0005
0
5
2.5
0
IL22
0.005
0.0025
0
5
2.5
0
IL27
0.0005
0.00025
0
5
2.5
0
IL10RB
0.05
0.025
0
9.02
4.51
0
ICOS
0.013
0.0065
0
5.3
2.65
0
IFNG
0.011
0.0055
0
5.3
2.65
0

Factors inducing lineage:

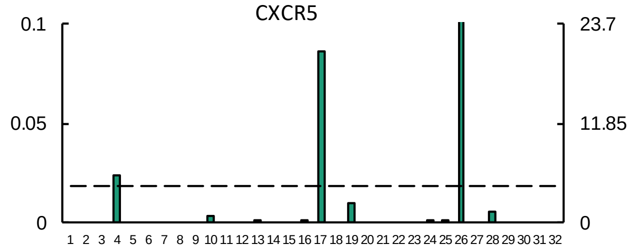

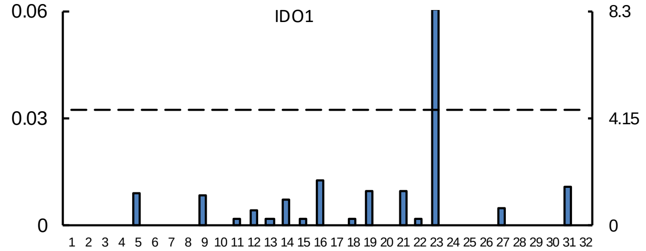

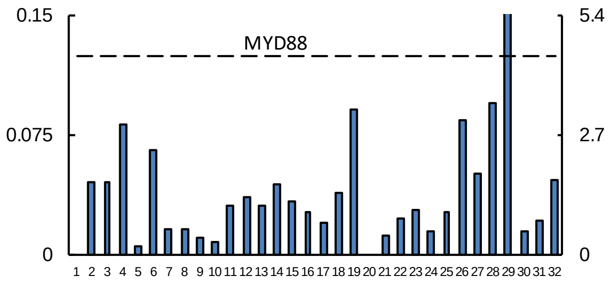

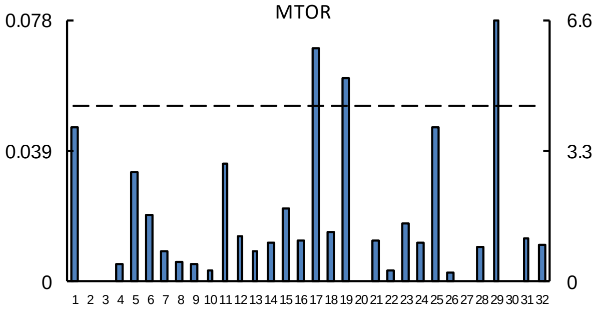

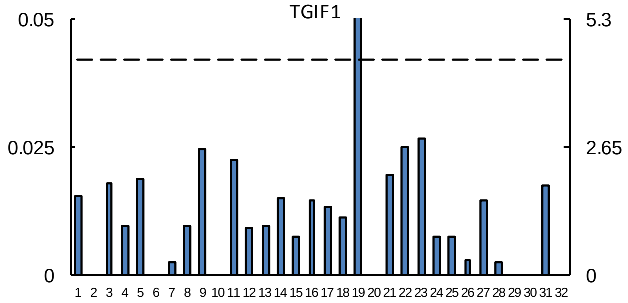

Receptor:

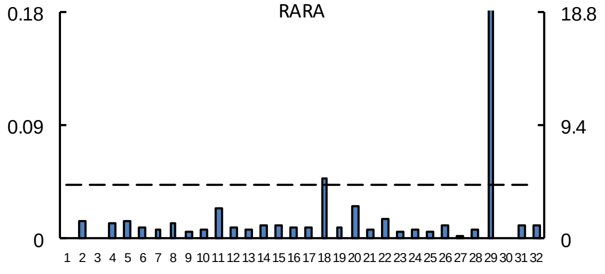

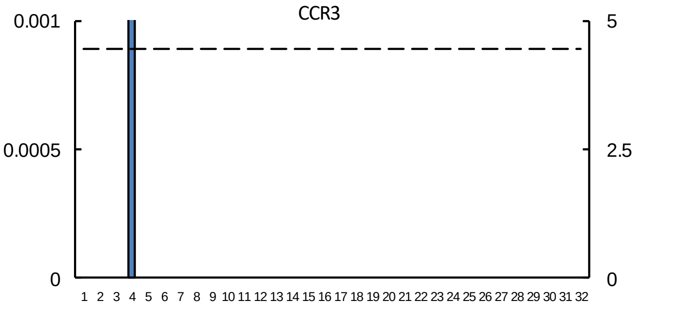

Continued:

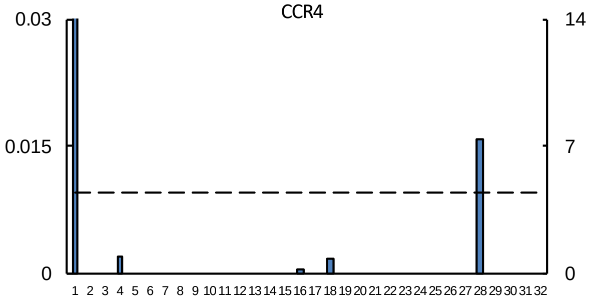

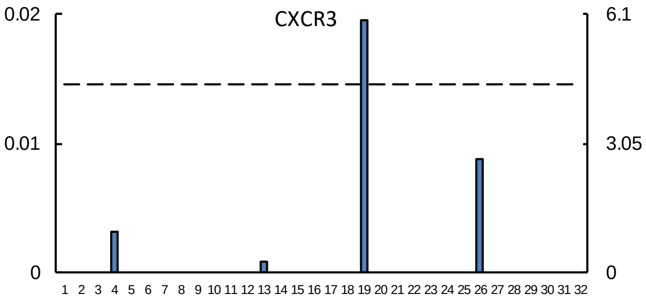

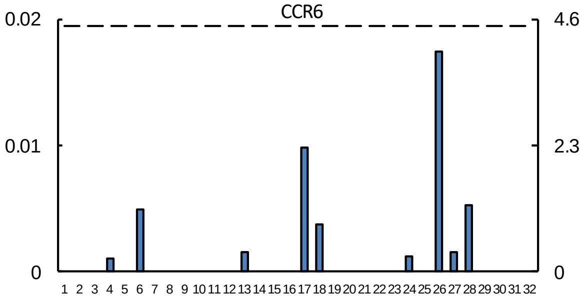

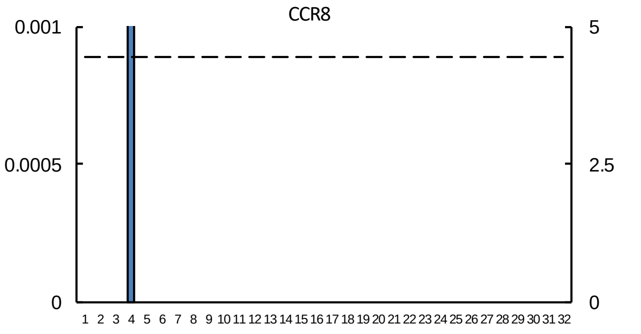

Others:

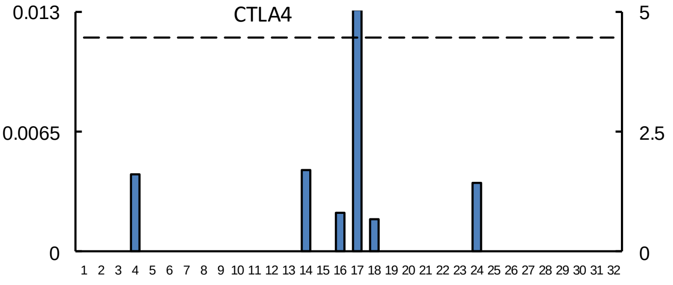

Supplemental figure 2. Tfh transcription factor (TF) Bcl6 and Th2-TF Gata3 nhibit, but Hdac6 promotes Treg to become APC-like Treg. .

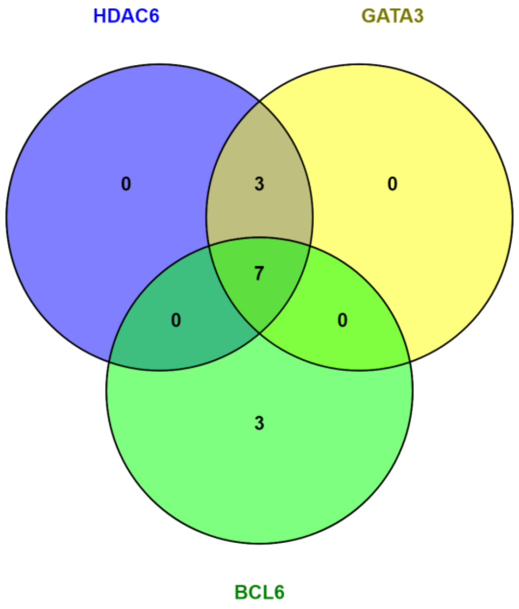

3 common signaling pathways that are shared in "HDAC6-/- downregulated" and "GATA3-/- upregulated":

1 Calcium-induced T Lymphocyte Apoptosis

2 IL-4 Signaling

3 Altered T Cell and B Cell Signaling in Rheumatoid Arthritis

7 common elements are shared in "HDAC6-/- downregulated", "GATA3-/- upregulated" and "BCL6-/- upregulated":

1 Antigen Presentation Pathway

2 B Cell Development

3 Autoimmune Thyroid Disease Signaling

4 Graft-versus-Host Disease Signaling

5 Nur77 Signaling in T Lymphocytes

6 T Helper Cell Differentiation

7 Allograft Rejection Signaling

3 elements are included exclusively in "BCL6-/- upregulated":

1 iCOS-iCOSL Signaling in T Helper Cells

2 Th2 Pathway

3 Th1 and Th2 Activation Pathway

Metabolic Cardiovascular Diseases (Supplemental Tables 1-6)

Supplemental Table 1. Insulin resistance inhibits the expression of Treg and Th22 regulators but promotes the other Th subset regulators in visceral adipose tissue of morbidly obsess patients.

Number of genes with significant expression changes*					The Number changes of gene list	up	3	2	1	3	1	1	
						down						1	1
Geo Dataset ID	Disease	Tissue	Sample definition	group		Gene. Symbol	TH1	TH2	TH9	TFH	TH17	Treg	TH22
GSE15773	Obesity	Omental adipose	Insulin resistant Vs. insulin sensitive	Transcription factor	7	AHR							-1.44
						FOXO1						-1.37	
						GATA3		1.46					
						IRF4		1.20	1.20	1.20			
						STAT1	1.44						
				cytokines	6	IL6				7.37	7.37		
						IL12RB1	1.27						
						ICOS				1.28			
						IFNG	1.73						
				immunoglobulin		CTLA4						1.40	

Number of genes with significant expression changes					The Number changes of gene list	up	2	1	1	1	1		
						down			2		1	3	
Geo Dataset ID	Disease	Tissue	Sample definition	Group		Gene symbol	TH1	TH2	TH9	TFH	TH17	Treg	TH22
GSE15773	Obesity	Subcutaneous adipose	Insulin resistant patient Vs. insulin sensitive patient	Transcription Factor	7	CMIP		1.38					
						MAF				1.24			
						RORA					-1.33		
						RORC					1.20		
						RUNX3	1.37						
				Transcription activators		STAT5A						-1.25	
						STAT6			1.18				
				CYtokines	5	IFNG	1.48						
				Factors inducing lineage		MTOR						-1.20	
				G protein-coupled receptors		CCR3			-1.21				
				Signal transducers and transcriptional modulators		SMAD2			-1.23			-1.23	

*For all the datasets, the 61 genes in table 1 were all examined. The genes with significant fold changes are shown in the table (P<0.05).

Supplemental Table 2. Type 2 diabetes and hyperlipidemia promote multiple Th subsets in Liver, adipose tissues, and spleen.

Number of genes with significant expression changes					The Number changes of gene list		TH1	TH2	TH9	TFH	TH17	Treg	TH22
						up	3		1		1	2	
						down			1	1	2	1	1
				group		Gene.symbol	TH1	TH2	TH9	TFH	TH17	Treg	TH22
GSE64998	Type 2 diabetes	Liver	Obese type2-diabetic vs. non-obese metabolically healthy	Transcription factor	8	AHR							-1.47
						FOXP3						1.16	
						MAF				-1.14			
						RORA					-1.34		
						RUNX3	1.21						
						SPI1			1.26				
						TBX21	1.25						
				transcription activators	5	STAT4	1.32						
				Factors inducing lineage		MYD88					1.23		
						MTOR						1.18	
						TGIF1			-1.23		-1.23	-1.23	

Number of genes with significant expression changes					The Number changes of gene list		TH1	TH2	TH9	TFH	TH17	Treg	TH22	TH25
						up	3	1	2	4	2	4	1	
						down					1	1		
				group		Gene.symbol	TH1	TH2	TH9	TFH	TH17	Treg	TH22	TH25
GSE76428	Type2 diabetes	Parametrial and periovarian adipose tissues	ApoE mice western diet Vs. Dhet mice western diet	Transcription factor	8	Ahr							1.48	
						Bach2						1.34		
						Eomes	1.18							
						Foxo1						1.46		
						Maf				1.66				
						Rora					-1.51			
				Transcription activators		Stat1	1.34							
						Stat3				1.62				
				Cytokines	11	Il5		1.41	1.41					
						Il6				1.50	1.50			
						Icos				1.62				
						Ifng	1.75							
				Factors inducing lineage		Mtor						-1.18		
						Tgif1			2.07		2.07	2.07		
				Antigen		Cd28						1.27		

Number of genes with significant expression changes				The Number changes of gene list		TH1	TH2	TH9	TFH	TH17	Treg	TH22
					up	3	1	2	4	2		1
					down				1			
			group		Gene.symbol	TH1	TH2	TH9	TFH	TH17	Treg	TH22
GSE6813	Spleen	CD4+CD25- Tconv cells from NOD(nonobese diabetic) mouse spleens Vs. B6 H2g7 mouse	Transcription factor	8	Ahr							1.30
					Irf4		1.80	1.80	1.80			
					Maf				2.66			
					Rora					1.28		
			Transcription activators		Stat1	1.40						
					Stat3				1.41			
			Cytokines	9	Il21				3.41	3.41		
					Icos				-4.89			
					Ifng	2.10						
			G protein-coupled receptor		Cxcr3	1.40		1.40				

Supplemental Table 3. Rosiglitazone (PPARγ agonist) induced failing hearts in apolipoprotein E-deficient mice significantly increased the expression of Th subset regulators

Number of genes with significant expression changes					The Number changes of gene list	up	2	1	4	4	4	5	1
						down							
				group		Gene.symbol	TH1	TH2	TH9	TFH	TH17	Treg	TH22
GSE28031	Atherosclerosis	Falling heart	Failing heart_apoE deficient 2 months of rosiglitazone Vs. control heart	Transcription factor	9	Ahr							2.72
						Cmip		2.56					
						Foxo1						2.92	
						Foxp3						2.93	
						Maf				2.49			
						Rora					2.65		
						Spi1			2.65				
				Transcription activators		Stat1	5.62						
						Stat3				3.78			
				cytokines	12	Il6				14.96	14.96		
				Factors inducing lineage		Cxcr5				2.44			
						Mtor						2.44	
						Tgif1			2.19		2.19	2.19	
				G protein-coupled receptor		Cxcr3	3.67		3.67				
				Beta chemokine receptor		Ccr6					4.48		
				Signal transducers and transcriptional modulators		Smad2			2.38			2.38	

Supplemental Table 4. Hyperlipidemia in ApoE-/- mice promote Th subset gene expressions in Aortic adventitia.

Number of genes with significant expression changes				up	1	1	4	3	3	1	2
				down		2	1	3	1	2	1
			group	Gene.symbol	TH1	TH2	TH9	TFH	TH17	Treg	TH22
GSE40156	AORTA	Atherosclerotic lesion of abdominal aorta Vs. adventitia of abdominal aorta	Transcription factor	Ahr							-1.41
				Batf				2.96			
				Bcl6				-1.72			
				Cmip		2.01					
				Foxo1						-1.60	
				Gata3		-4.38					
				Irf4		-7.48	-7.48	-7.48			
				Rorc					-32.96		
				Spi1			6.53				
			Transcription activators	Stat3				1.93			
				Stat6			1.65				
			Cytokines	Ccl17							4.78
				Il6				27.99	27.99		
				Il10rb							3.82
				Icos				-4.89			
			Factors inducing lineage	Myd88					1.66		
				Mtor						-2.78	
				Tgif1			5.05		5.05	5.05	
			G protein-coupled receptor	Cxcr3	2.12		2.12				

Dataset	Tissue	Comparison	group	Gene.symbol	TH1	TH2	TH9	TFH	TH17	Treg	TH22
Number of genes with significant expression changes				up	6	2	5	8	5	5	2
				down			1	1	1	1	
GSE40156	Adventitia with ATLO of abdominal aorta within atherosclerotic lesion area	ApoE- adv. lesion vs. WT-adventitia	Transcription factor	Batf				3.81			
				Bcl6				-1.88			
				Eomes	2.30						
				Foxo3						1.56	
				Foxp3						2.55	
				Irf4		2.25	2.25	2.45			
				Maf				5.73			
				Rorc					-5.59		
				Spi1			3.42				
				Tbx21	3.26						
			Transcription activators	Stat1	2.67						
				Stat3				1.95			
				Stat4	17.01						
				Stat6			1.56				
			Cytokines	Ccl17							1.56
				Il6				17.06	17.06		
				Il9			-2.15				
				Il21				3.04	3.04		
				Il10rb							1.82
				Icos				5.39			
			Factors inducing lineage 46	Cxcr5				3.72			
				Myd88					2.29		
				Mtor						-1.74	
				Tgif1			1.56		1.56	1.56	
			Retinoic acid receptor	Rara	4.11						
			G protein-coupled receptor	Cxcr3	5.25		5.25				
			Beta chemokine receptor	Ccr6					4.48		
			Antigen	Cd28						6.29	
			Immunoglobulin	Ctla4						4.41	
			Transcription repressor	Gfi1		4.00					

Dataset	Tissue	Comparison	group	Gene.symbol	TH1	TH2	TH9	TFH	TH17	Treg	TH22
Number of genes with significant expression changes				up	1	1	1				1
				down							
GSE40156	Adventitia with ATLO of abdominal aorta outside atherosclerotic lesion area	ApoE- adventitia vs. WT-adventitia	Transcription factor	Ahr							1.77
			cytokines	Il13		5.35					
			G protein-coupled receptor	Cxcr3	1.40		1.40				

Supplemental Table 5. Aged ApoE -/- mice blood express fewer Th subset regulators than wild-type (WT) mouse blood.

Dataset	Tissue	Comparison	group	Gene.symbol	TH1	TH2	TH9	TFH	TH17	Treg	TH22
Number of genes with significant expression changes				up			2	1	2		1
				down		1	1	2		1	
GSE40156	Blood	ApoE-Blood-78wks Vs. WT-Blood-78 wks	Transcription factor	Ahr							1.69
				Foxo1						-1.52	
				Irf4		-1.37	-1.37	-1.37			
				Rora					1.34		
				Spi1			1.75				
			Transcription activators	Stat3				1.47			
				Stat6			1.34				
			Factors inducing lineage	Cxcr5				-1.58			
				Myd88					1.43		

Supplemental Table 6. CD3+ T cells from patients with familiar hypercholesterolemia (FH) express Th subset genes except Th1.

Number of genes with significant expression changes					TH1	TH2	TH9	TFH	TH17	Treg	TH22	TH25
				up		1		1		1		
				down			1			1		
			group	Gene.symbol	TH1	TH2	TH9	TFH	TH17	Treg	TH22	TH25
GSE6088	Human CD3 positive Tcells	Homozygot FH Vs. Control participant	Transcription factor	FOXP3						-3.75		
				GATA3		1.81						
				MAF				2.07				
			Transcription activators	STAT6			-1.49					
			Immunoglobulin	CTLA4						1.66		

Number of genes with significant expression changes					TH1	TH2	TH9	TFH	TH17	Treg	TH22	TH25
				up		1	1	2		2	1	
				down	1		1	1	2	1		
			group	Gene.symbol	TH1	TH2	TH9	TFH	TH17	Treg	TH22	TH25
GSE6088	Human CD3 positive T cells	Heterozgot FH Vs. Control participant	Transcription factor	AHR							1.39	
				IKZF4						1.34		
				RORA					-2.30			
				RUNX3	-1.94							
			Cytokines	IL4		2.20	2.20					
				IL21				-1.98	-1.98			
				ICOS				1.69				
			Factors inducing lineage	CXCR5				1.75				
			Signal transducers and transcriptional modulators	SMAD3			-1.27			-1.27		
			Immunoglobulin	CTLA4						1.76		

Autoimmunity diseases (supplemental Tables 7-9)

Supplemental Table 7. Lupus Erythematosus increases Th2, Th9,TFH subsets but decreases Th1 and Treg in patients' blood.

Number of genes with significant expression changes					The Number changes of gene list									
						up	3	5	5	5	4	4	1	1
						down	6	2	4	3	4	8		
				group		Gene.symbol	TH1	TH2	TH9	TFH	TH17	Treg	TH22	TH25
GSE61635	lupus erythematosus	WHOLE BLOOD	SLE Patient Vs. healthy people	Transcription Factor	22	BATF				1.13				
						BACH2						-2.38		
						BCL6				1.57				
						CMIP		1.28						
						EOMES	-1.85							
						FOXO3						-2.45		
						FOXP3						1.10		
						GATA3		-1.86						
						TBX21	-1.66							
						STAT1	2.16							
						FOXO1						-1.57		
						IRF4		1.11	1.11	1.11				
						MAF				-1.18				
						RORC					-1.71			
						RORA					-1.88			
						RUNX3	-1.51							
						SPI1			1.54					
				Transcription activators		STAT3				1.56				
						STAT4	-1.68							
						STAT5A						1.13		
				cytokines	33	IL2	1.38	1.38	1.38			1.38		
						IL4		-1.61	-1.61					
						IL10RB							1.36	
						IL12RB1	-1.19							
						IL17F					1.14			
						IL21				1.83	1.83			
						IL22					1.36			
						IL25								1.11
						ICOS				-1.76				
				Factors inducing lineage		CXCR5				-2.53				
						IDO1		2.17						
						MYD88					1.45			
						MTOR						-1.24		
						TGIF1			-1.81		-1.81	-1.81		
				retinoic acid receptor		RARA	1.45							
				signal transducers and transcriptional modulators		SMAD2			1.13			1.13		
						SMAD3			-1.43			-1.43		
				G protein-coupled receptors		CCR3			1.44					
						CXCR3	-1.61		-1.61					
				beta chemokine receptor		CCR6					-2.00			
						CCR8		1.27						
				Antigen immunoglobulin		CD28						-1.98		
						CTLA4						-1.46		

Supplemental Table 8. Interferon-gamma (IFN-Y) therapy in lupus patients increases more Th1 and Th17 subsets than non-treatment controls in blood cells.

Number of genes with significant expression changes					The Number changes of gene list	up	5	4	4	4	5	5	1
						down	4	1	4	3	2	6	
				group		Gene.symbol	TH1	TH2	TH9	TFH	TH17	Treg	TH22
GSE72754	Lupus Erythematosus	whole blood	SLE Patient IFN-K Vs. Healthy individual, no treatment	Transcription Factor	19	BATF				1.30			
						BACH2						-2.33	
						EOMES	-2.10						
						FOXO1						-1.86	
						FOXP3						1.28	
						GATA3		-1.75					
						HNF1A				1.25	1.25		
						IRF4		1.18	1.18	1.18			
						IKZF4						1.19	
						MAF				-1.81			
						RORA					-2.36		
						RUNX3	-1.76						
						TBX21	-1.51						
				Transcription activators		STAT1	1.80						
						STAT5A						-1.13	
						STAT6			-1.34				
				Cytokines	29	CCL17							1.19
						IL2	1.10	1.10	1.10			1.10	
						IL5		1.22	1.22				
						IL12RB1	1.32						
						IL17B					1.23		
						IL21				1.16	1.16		
						IL22					1.24		
						IL27	1.39						
				Factors inducing lineage		CXCR5				-1.77			
						TGIF1			1.22		1.22	1.22	
				Retinoic acid receptor		RARA	1.24						
				G protein-coupled receptors		CCR3			-2.57				
						CXCR3			-1.28			-1.28	
				Signal transducers and transcriptional modulators		SMAD2					-2.31		
				Beta chemokine receptor		CCR6			-1.77			-1.77	
				Signal transducers and transcriptional modulators		SMAD3						-1.59	
				Antigen		CD28						1.34	
				Immunoglobulin		CTLA4		1.34					
				Transcriptional repressor		GFI1	-1.22			-1.22			

Supplemental Table 9. Psoriasis in patients induces all Th subsets except Treg in skin.

Number of genes with significant expression changes					The Number changes of gene list	up	5	4	4	4	5	5	1
						down	4	1	4	2	2	6	
				group		Gene.symbol	TH1	TH2	TH9	TFH	TH17	Treg	TH22
GSE52471	Psoriasis	Skin biopsy	psoriasis Vs. normal	Transcription Factor	19	BATF				1.30			
						BACH2						-2.33	
						EOMES	-2.10						
						FOXO1						-1.86	
						FOXP3						1.28	
						GATA3		-1.75					
						HNF1A				1.25	1.25		
						IRF4		1.18	1.18	1.18			
						IKZF4						1.19	
						MAF				-1.81			
						RORA					-2.36		
						RUNX3	-1.76						
						TBX21	-1.51						
				Transcription activators		STAT1	1.80						
						STAT5A						-1.13	
						STAT6			-1.34				
				CYtokines	28	CCL17							1.19
						IL2	1.10	1.10	1.10			1.10	
						IL5		1.22	1.22				
						IL12RB1	1.32						
						IL17B					1.25		
						IL21				1.16	1.16		
						IL22					1.24		
						IL27	1.39						
				Factors inducing lineage		CXCR5				-1.77			
						TGIF1			1.22		1.22	1.22	
				Retinoic acid receptor		RARA	1.24						
				G protein-coupled receptors		CXCR3	-1.22		-1.22				
				Beta chemokine receptor		CCR6					-2.31		
				Signal transducers and transcriptional modulators		SMAD2			-1.28			-1.28	
						SMAD3			-1.77			-1.77	
				Antigen		CD28						-1.59	
				Transcriptional repressor		GFI1		1.34					
				Immunoglobulin		CTLA4						1.34	

Physical Injury (Supplemental Tables 10-11)

Supplemental Table 10. Severe Trauma in patients increases Th2,Th9,TFH, and Th17 subsets but decreases Th1 and Treg in blood cell populations.

Number of genes with significant expression changes					The Number changes of gene list									
						up	3	5	5	6	6	6	1	1
						down	7	3	4	2	3	7	1	
				group		Gene.symbol	TH1	TH2	TH9	TFH	TH17	Treg	TH22	TH25
GSE36809	Injury	White Blood Cells	Severe Trauma Injury Vs. control	Transcription factor	27	AHR							-1.99	
						BATF				2.68				
						BACH2						-5.81		
						BCL6				10.69				
						CMIP		-1.16						
						EOMES	-5.25							
						FOXO1						-2.02		
						FOXO3						1.34		
						FOXP3						1.26		
						GATA3		-7.03						
						HNF1A				1.36	1.36			
						IRF4		1.12	1.12	1.12				
						IKZF4						-1.54		
						MAF				-3.77				
						RORC					-1.61			
						RORA					-5.82			
						RUNX3	-3.09							
						SPI1			6.22					
						TBX21	-2.17							
				Transcription activators		STAT1	-1.82							
						STAT3				3.63				
						STAT4	-3.78							
						STAT5A						1.62		
						STAT6			3.64					
				Cytokines	33	IL2	1.13	1.13	1.13			1.13		
						IL4		-1.17	-1.17					
						IL12RB1	1.41							
						IL13		1.22						
						IL17B					1.13			
						IL17F					1.15			
						IL22					1.10			
						IL25								1.18
						IL10RB							2.82	
						ICOS				-2.61				
						IFNG	-1.48							
				Factors inducing lineage		CXCR5				1.42				
						MYD88					1.73			
						MTOR						1.16		
						TGIF1			1.40		1.40	1.40		
				Retinoic acid receptor		RARA	3.64							
				G-protein-coupled receptor		CCR4		1.24						
						CXCR3	-1.84		-1.84					
				Beta chemokine receptor		CCR6					-2.30			
						CCR8		1.24						
				Signal transducers and transcriptional modulators		SMAD2			-1.45			-1.45		
						SMAD3			-2.12			-2.12		
				Antigen		CD28						-6.37		
				Immunoglobulin		CTLA4						-1.41		

Supplemental Table 11. Burn Injury in patients decreases all the Th1 subset regulators except that of Th22 in blood cells.

Number of genes with significant expression changes					The Number changes of gene list									
						up	1	3	3	3	1	2	1	
						down	8	5	6	3	6	9	1	
				group		Gene.symbol	TH1	TH2	TH9	TFH	TH17	Treg	TH22	TH25
GSE37069	Injury	White Blood Cells	Burn Injury Vs. control	Transcription factor	21	AHR							-1.35	
						BATF				1.78				
						BACH2						-1.86		
						BCL6				3.41				
						CMIP		1.33						
						EOMES	-4.68							
						FOXO1						-1.74		
						FOXP3						-1.25		
						GATA3		-4.31						
						IKZF4						-1.18		
						MAF				-2.49				
						RORC					-2.10			
						RORA					-6.73			
						RUNX3	-2.73							
						SPI1			2.39					
						TBX21	-2.77							
				Transcription activators		STAT1	-2.42							
						STAT3				2.02				
						STAT4	-3.23							
						STAT5A						1.18		
						STAT6			1.55					
				cytokines	32	IL4		-1.14	-1.14					
						IL5		-1.09	-1.09					
						IL12RB1	-1.49							
						IL13		-1.19						
						IL17B					-1.15			
						IL17F					-1.14			
						IL22					1.10			
						IL10RB							2.07	
						ICOS				-1.88				
						IFNG	-2.03							
				Factors inducing lineage		CXCR5				-1.19				
						IDO1		-2.00						
						MYD88					1.45			
						MTOR						-1.14		
						TGIF1			-1.65		-1.65	-1.65		
				Retinoic acid receptor		RARA	1.38							
				G protein-coupled receptors		CCR3			-3.34					
						CCR4		1.12						
						CXCR3	-2.22		-2.22					
				Beta chemokine receptor		CCR6					-1.81			
						CCR8		1.12						
				Signal transducers and transcriptional modulators		SMAD2			1.22			1.22		
						SMAD3			-2.04			-2.04		
				Antigen		CD28						-4.08		
				Immunoglobulin		CTLA4						-1.18		

Vascular cell cytokines, as innate immune cells, promote Th subset development by secreting Th subset increasing Cytokines (Supplemental Tables 12-13).

Supplemental Table 12. Aortic endothelial cells activated by pro-atherogenic stimuli upregulated cytokine genes that promote more effector Th subsets than Treg.

Number of genes with significant expression changes						TH1	TH2	TH9	TFH	TH17	Treg	TH22
					up		2	2	2	1		1
					down							
GSE39264	Atherosclerosis	Aortic endothelial cells	DMEM+LPS, 4hrs Vs. DMEM cell media (atherosclerotic treatment)	group	Gene.symbol	TH1	TH2	TH9	TFH	TH17	Treg	TH22
				Cytokines	Ccl17							1.19
					Il4		1.22	1.22				
					Il6				2.19	2.19		
					Il9			1.35				
					Il13		1.25					
					Icos				1.28			

Number of genes with significant expression changes						TH1	TH2	TH9	TFH	TH17	Treg	TH22
					up	1	2	3			1	
					down							1
GSE39264	Atherosclerosis	Aortic endothelial cells	DMEM + oxLDL, 4hrs Vs. DMEM	group	Gene.symbol	TH1	TH2	TH9	TFH	TH17	Treg	TH22
				cytokines	Il2	1.30	1.30	1.30			1.30	
					Il5		1.17	1.17				
					Il9			1.25				
					Il10rb							-1.55

Number of genes with significant expression changes						TH1	TH2	TH9	TFH	TH17	Treg	TH22
					up	1	1	2			1	
					down				1	1		1
GSE39264	Atherosclerosis	Aortic endothelial cells	oxPAPC Vs. DMEM	group	Gene.symbol	TH1	TH2	TH9	TFH	TH17	Treg	TH22
				cytokines	Il2	1.26	1.26	1.26			1.26	
					Il6				-4.80	-4.80		
					Il9			1.20				
					Il10rb							-2.34

Supplemental Table 13. Vascular smooth muscle cells in response to pro-inflammatory stimuli, upregulate cytokine genes that promote more Th1, Th9, TFH, Th17 than Treg.

Number of genes with significant expression changes				up					2	1			
				down									
GSE15062	Atherosclerosis	Aortic Smooth Muscle Cells	mSMC-TNF Vs. mSMC-Control	group	Gene.symbol	TH1	TH2	TH9	TFH	TH17	Treg	TH22	TH25
				cytokines	Il6				2.30	2.30			
					Icos				1.47				

Numbers of gene expression changes				up					1	1			
				down									
GSE15062	Atherosclerosis	Aortic Smooth Muscle Cells	mSMC-TNF-LTBR Vs. mSMC	group	Gene.symbol	TH1	TH2	TH9	TFH	TH17	Treg	TH22	TH25
				cytokines	Il6				1.94	1.94			

Number of genes with significant expression changes				up		1							
				down									
GSE68021	Atherosclerosis	Human Vascular smooth muscle cells	Native LDL endocytosis Vs. VSMC control	group	Gene.symbol	TH1	TH2	TH9	TFH	TH17	Treg	TH22	TH25
				cytokines	IL27	1.40							

Number of genes with significant expression changes					up				1	1			
					down		1	1	1	2			1
GSE83500	Myocardial infraction	Vascular smooth muscle cells	MI patient Vs. non-Mi patient	group	Gene.symbol	TH1	TH2	TH9	TFH	TH17	Treg	TH22	TH25
				cytokines	IL4		-1.2	-1.2					
					IL6				-1.3	-1.3			
					IL17F					-1.28			
					IL21				1.21	1.21			
					IL25								-1.20

Number of genes with significant expression changes					up				1	1		
					down		1	1				
GSE30638	Atherosclerosis	Aorta	LDLR-KO mice western-type diet, sham treatment Vs. LDLR-KO, chow diet, sham treatment	group	Gene.symbol	TH1	TH2	TH9	TFH	TH17	Treg	TH22
				cytokines	Il4		-2.78	-2.78				
					Il6				6.14	6.14		

Several Th subset-associated transcription factors and regulators such as GATA3, Bcl6 and Hdac6 regulate the expression of Th subset regulators in Treg, suggesting that these transcription regulators modulate Treg plasticity (Supplemental Tables 14-21).

Supplemental Table 14. Diabetes "swing" Treg to be plastic and express other Th subset genes in Treg.

Number of genes with significant expression changes				up	2		1	3	2		
				down				1		1	
			group	Gene.symbol	TH1	TH2	TH9	TFH	TH17	Treg	TH22
GSE6813	Spleen	CD4+CD25+Treg cells from NOD(nonobese diabetic) mouse spleens Vs. B6H2g7mouse spleens	Transcriptional factor	Bach2						-1.25	
				Bcl6				1.29			
				Eomes	1.91						
				Maf				2.13			
				Rora					1.32		
				Runx3	1.45						
			Transcription activators	Stat3				1.31			
			Cytokines	Icos				-3.87			
			G protein-coupled receptors	Ccr3			1.23				
			Beta chemokine receptor	Ccr6					1.35		

Supplemental Table 15. Induced Treg are less likely to express Th subset genes except interferon-gamma (IFNγ) than natural Treg, suggesting that induced Treg have less ability to convert in to other Th subsets compared to natural Treg. Induced Treg have higher potential than natural Treg to become IFNγ-secreting Th1-like Treg.

Number of genes with significant expression changes				up	1				1	2	
				down		1	2	3	2	3	1
			group	Gene.symbol	TH1	TH2	TH9	TFH	TH17	Treg	TH22
GSE35543	Spleens and mesenteric lymph nodes	Induced Treg (iTreg) Vs. Natural Treg(nTreg)	Transcription factor	Ahr							-1.73
				Batf				-1.37			
				Bach2						2.04	
				Bcl6				-1.66			
				Ikzf4						-2.71	
				Rorc					1.69		
				Rora					-3.16		
			Cytokines	Icos				-1.60			
				Ifng	1.53						
			Factors inducing lineage	Mtor						1.53	
				Tgif1			-1.81		-1.81	-1.81	
			G protein-coupled receptors	Ccr3			-1.97				
			Beta chemokine receptor	Ccr8		-2.69					
			Immunoglobulin	Ctla4						-1.40	

Number of genes with significant expression changes				up					1		1	
				down								
GSE19512	Spleen and lymph nodes	Mouse in vivo iTreg cells Vs. Mouse in vivo nTreg	group	Gene.symbol	TH1	TH2	TH9	TFH	TH17	Treg	TH22	TH25
			Transcription factor	Ahr							1.52	
				Rorc					1.25			

Supplemental Table 16. Deficiency of Foxp3 mutation in Scurfy mice upregulate other TH subset regulators in CD4+ T cell populations.

Number of genes with significant expression changes						TH1	TH2	TH9	TFH	TH17	Treg	TH22
					up	7	7	6	7	3	6	2
					down	1		1			4	1
				group	Gene.symbol	TH1	TH2	TH9	TFH	TH17	Treg	TH22
GSE11775	Scurfy mutation	Lymph node cells	Effector CD4+ T cells from scurfy mice Vs. effortor CD4 Healthy B6	Transcription factor	Ahr							5.75
					Batf				2.37			
					Bach2						-4.83	
					Eomes	3.39						
					Foxo1						-3.01	
					Foxo3						-2.09	
					Foxp3						1.63	
					Gata3		2.41					
					Irf4		1.60	1.60	1.60			
					Ikzf4						1.90	
					Maf				3.55			
					Rora					2.64		
					Runx3	1.23						
					Spi1			1.63				
					Tbx21	6.55						
				Transcription activators	Stat1	-1.54						
					Stat4	1.24						
					Stat6			-1.71				
				Cytokines	Ccl17							2.11
					Il2	1.86	1.86	1.86			1.86	
					Il4		15.57	15.57				
					Il6				1.90	1.90		
					Il13		2.84					
					Il10rb							-1.33
					Il21				1.30	1.30		
					Icos				3.98			
					Ifng	9.61						
				Factors inducing lineage	Cxcr5				2.67			
					Mtor						1.44	
				G-protein-coupled receptor	Ccr4		1.32					
					Cxcr3	4.58		4.58				
				Beta chemokine receptor	Ccr8		1.57					
				Signal transducers and transcriptional modulators58	Smad3			1.44			1.44	
				Antigen	Cd28						-1.60	
				Immunoglobulin	Ctla4						8.23	

Number of genes with significant expression changes						TH1	TH2	TH9	TFH	TH17	Treg	TH22
					up	4	3	2	1	1	1	2
					down			2	1	3	5	1
				group	Gene.symbol	TH1	TH2	TH9	TFH	TH17	Treg	TH22
GSE11775	Scurfy mutation	Lymph node cells	Treg Foxp3-deficient CD4+Cells from Scurfy mice Vs. Foxp3-sufficient Treg CD4 cells from healthy B6	Transcription factor	Ahr							1.86
					Bach2						-3.63	
					Eomes	3.01						
					Foxo1						-1.94	
					Foxo3						-1.78	
					Foxp3						-5.84	
					Rorc					-1.61		
					Tbx21	3.94						
				Transcription activators	Stat6			-1.69				
				Cytokines	Ccl17							1.74
					Il2	2.28	2.28	2.28			2.28	
					Il4		13.9	13.9				
					Il13		2.18					
					Il21				1.52	1.52		
					Il10rb							-2.01
					Ifng	3.10						
				Factors inducing lineage	Cxcr5				-1.41			
					Tgif1			-1.83		-1.83	-1.83	
				Beta chemokine receptor	Ccr6					-2.30		

Number of genes with significant expression changes					TH1	TH2	TH9	TFH	TH17	Treg	TH22
				up	5	2	2	3	2	5	
				down	2	6	7	5	2	5	2
			group	Gene.symbol	TH1	TH2	TH9	TFH	TH17	Treg	TH22
GSE35543	Spleens and mesenteric lymph nodes	Lost Foxp3 expression (ex-iTreg) Vs. Natural Treg(nTreg)	Transcription factor	Ahr							-1.94
				Batf				-1.49			
				Bach2						1.50	
				Bcl6				1.74			
				Cmip		-1.51					
				Eomes	2.81						
				Foxo1						-1.59	
				Foxo3						-1.16	
				Gata3		-2.14					
				Irf4		-2.04	-2.04	-2.04			
				Ikzf4						-14.72	
				Maf				-1.47			
				Rorc					1.29		
				Rora					-1.92		
				Tbx21	1.38						
			Transcription activators	Stat1	-1.79						
				Stat3				-2.01			
				Stat4	1.25						
				Stat6			-1.24				
			Cytokines	Il2	9.78	9.78	9.78			9.78	
				Il4		2.22	2.22				
				Il5		-1.16	-1.16				
				Il21				38.59	38.59		
				Il10rb							-1.52
				Icos				-1.55			
				Ifng	3.78						
			Factors inducing lineage	Cxcr5				1.26			
				Mtor						1.17	
				Tgif1			-1.46		-1.46	-1.46	
			G protein-coupled receptors	Ccr3			-4.96				
				Ccr4		-1.33					
				Cxcr3	-1.39		-1.39				
			Beta chemokine receptor	Ccr8		-3.29					
			Signal transducers and transcriptional modulators	Smad3			-1.39			-1.39	
			Antigen	Cd28						1.40	
			Immunoglobulin	Ctla4						1.40	

Supplemental Table 17. CD4+ T cells in Foxo3 Scurfy mice require IL-2 to express some Th subset regulators.

Number of genes with significant expression changes					TH1	TH2	TH9	TFH	TH17	Treg	TH22
				up	2	3	1	2	1	2	
				down		3	1	1	1	2	2
			group	gene symbol	TH1	TH2	TH9	TFH	TH17	Treg	TH22
GSE23398	FACS sorted CD4+ Tcells Scurfy	Scurfy(B6.Cg-Foxo3sf/J Vs. Scurfy mice with a null IL-2 gene sf.il2-/-)	Transcription factor	Ahr							-2.89
				Batf				1.78			
				Eomes	3.20						
				Foxp3						-3.61	
				Ikzf4						-3.66	
				Rorc					1.67		
			Cytokines	Ccl17							-1.49
				Il2	3.43	3.43	3.43			3.43	
				Il5		-3.01	-3.01				
				Il6				-4.06	-4.06		
				Il13		-22.62					
			Factors inducing lineage	Cxcr5				4.06			
				Ido1		1.42					
			G-protein-coupled receptor	Ccr4		1.57					
			Beta chemokine receptor	Ccr8		-3.07					
			Antigen	Cd28						1.74	

Supplemental Table 18. MicroRNA processing enzyme Dicer inhibits other Th subset genes expression in Treg.

					TH1	TH2	TH9	TFH	TH17	Treg	TH22	TH25
Number of genes with significant expression changes				up	7	3	4	4	3	4	1	
				down		1		1	2	2	1	
			group	Gene.symbol	TH1	TH2	TH9	TFH	TH17	Treg	TH22	TH25
GSE11818	CD4+YFP+Tcells	Tcells_FoxP3-GFP-hCre ROSA26R-YFP Dicer/lox(KO) Vs. cells_FoxP3-GFP-hCre ROSA26R-YFP Dicer/lox(Het)	Transcription factor	Ahr							-1.18	
				Batf				1.40				
				Bach2						3.36		
				Bcl6				-1.21				
				Eomes	7.62							
				Foxo1						-1.37		
				Foxo3						-1.25		
				Rora					-1.67			
				Runx3	2.36							
				Tbx21	4.38							
			Transcription activators	Stat1	1.88							
				Stat3				1.54				
			Cytokines	Il2	1.79	1.79	1.79			1.79		
				Il4		11.4	11.4					
				Il13		2.69						
				Il21				2.95	2.95			
				Il10rb							1.51	
				Icos				1.37				
				Ifng	4							
			Factors inducing lineage	Myd88					1.21			
				Mtor						1.31		
			G-protein-coupled receptor	Ccr4		-1.24						
				Cxcr3	2.62		2.62					
			Beta chemokine receptor	Ccr6					-3.58			
			Signal transducers and transcriptional modulators	Smad2			1.19			1.19		
			Immunoglobulin	Ctla4					1.70			

Supplemental Table 19. Xbp1 promotes Treg and Th2 regulators but inhibits the expression of other Th subset regulators

					TH1	TH2	TH9	TFH	TH17	Treg	TH22	TH25
Number of genes with significant expression changes				up	3	3	5	2	6	3	1	
				down	1	5	1			2		1
			group	Gene.symbol	TH1	TH2	TH9	TFH	TH17	Treg	TH22	TH25
GSE40273	Spleen	CD4+CD25hi Tregs, Xbp1 Knockout Vs. CD4+CD25hi Tregs, wild type	Transcription factor	Ahr							1.27	
				Cmip		-1.09						
				Foxo3						-1.11		
				Gata3		-1.17						
				Hnf1a				1.10	1.10			
				Rorc					1.16			
				Spi1			1.21					
				Tbx21	1.29							
			Transcription activators	Stat1	-1.22							
			Cytokines	Il2	1.49	1.49	1.49			1.49		
				Il5		1.23	1.23					
				Il13		1.17						
				Il17f					1.11			
				Il25								-1.10
			Factors inducing lineage	Cxcr5				1.17				
				Ido1		-1.16						
				Myd88					1.16			
				Tgif1			1.12		1.12	1.12		
			G protein-coupled receptor	Cxcr3	1.18		1.18					
			Beta chemokine receptor	Ccr6					1.10			
				Ccr8		-1.31						
			Signal transducers and transcriptional modulators	Smad3			-1.10			-1.10		
			Immunoglobulin	Ctla4						-1.19		
			Transcriptional repressor	Gfi1		-1.10						

Supplemental Table 20. GATA1 inhibits Treg to express other Th subset genes.

Number of genes with significant expression changes					TH1	TH2	TH9	TFH	TH17	Treg	TH22	TH25
				up	3		1	1	1			
				down								
			group	Gene.symbol	TH1	TH2	TH9	TFH	TH17	Treg	TH22	TH25
GSE40273	Spleen	CD4+CD25hi Tregs, Gata1 Knockout Vs. CD4+CD25hi Tregs, wild type	Transcription Factor	Eomes	1.24							
			Transcription activators	Stat1	1.30							
			Factors inducing lineage	Cxcr5				1.50				
			G protein-coupled receptor	Cxcr3	1.45		1.45					
			Beta chemokine receptor	Ccr6					1.29			

Supplemental Table 21. E. CTLA-4 inhibits Th subset gene expression but promotes IL-2 and other Treg gene expression.

Number of genes with significant expression changes					TH1	TH2	TH9	TFH	TH17	Treg	TH22	TH25
				up	1	1	1	5	2	1	1	1
				down	2	1	1			4	1	
			group	Gene.symbol	TH1	TH2	TH9	TFH	TH17	Treg	TH22	TH25
GSE37563	T cells	CTLA-4 KO T cell Vs. wild type Tcell	Transcription factor	Ahr							1.82	
				Batf				1.58				
				Bach2						-2.71		
				Bcl6				1.38				
				Foxo3						-3.59		
				Ikzf4						-1.72		
				Maf				1.55				
				Rora					1.66			
			Transcription activators	Stat1	-1.62							
			Cytokines	Ccl17							-2.88	
				Il2	-1.57	-1.57	-1.57					
				Il21				1.76	1.76			
				Il25								3.29
			Factors inducing lineage	Cxcr5				2.17				
				Mtor						1.33		
			G-protein-coupled receptor	Ccr4		1.76						
				Cxcr3	1.99		1.99					
			Immunoglobulin	Ctla4						-6.15		

Supplemental Table 22. MyD88 is required for some Th subset gene expression.

Number of genes with significant expression changes				up	1	1	1			1	1	
				down			2	2	3	1	1	
GSE25742	Whole blood	MYD88-/- Patient Vs. healthy control	group	Gene.symbol	TH1	TH2	TH9	TFH	TH17	Treg	TH22	TH25
			Transcription factor	AHR							-1.27	
				BCL6				-1.59				
				FOXO3						-1.35		
				IKZF4						6.65		
				RORA					-1.39			
				SPI1			-1.63					
			Transcription activators	STAT6			-1.27					
			Cytokines	IL6				-13.5	-13.5			
				IL10RB							1.59	
			Factors inducing lineage	IDO1		4.89						
				MYD88					-1.62			
			G protein-coupled receptor	CXCR3	3.54		3.54					

Supplemental Table 23. G. Treg-specific PPARγ INHIBITS Th subset gene expression in adipose tissue and lymph nodes.

Number of genes with significant expression changes				up	1	3	2	3	2	2	1	
				down			1	1				
GSE37532	lymph nodes	LN.Treg-Pparg.mut Vs. LN.Treg-Pparg.wt	group	Gene.symbol	TH1	TH2	TH9	TFH	TH17	Treg	TH22	TH25
			Transcription factor	Ahr							2.01	
				Bach2						-1.51		
				Foxo1						-1.30		
				Irf4		1.27	1.27	1.27				
				Maf				1.27				
				Rora					1.41			
			cytokines	Icos				1.19				
			Factors inducing lineage	Cxcr5				-1.29				
			G protein-coupled receptors	Ccr3			-1.48					
				Ccr4		1.38						
				Cxcr3	1.37		1.37					
			Beta chemokine receptor	Ccr6					1.58			
				Ccr8		1.57						
Number of genes with significant expression changes				up	6	4	5	5	5	6	1	
				down	1	3	2	1	1	1		
GSE37532	Visceral adipose tissue	VAT.B6.Treg-Pparg.mut Vs. VAT.B6.Treg-Pparg wt	group	Gene.symbol	TH1	TH2	TH9	TFH	TH17	Treg	TH22	TH25
			Transcription factor	Ahr							2.57	
				Bach2						3.45		
				Bcl6				1.58				
				Foxo1						1.45		
				Foxo3						-1.32		
				Gata3		-1.35						
				Irf4		-1.92	-1.92	-1.92				
				Ikzf4						1.75		
				Rorc					1.21			
				Rora					-1.29			
				Tbx21	2.34							
			Transcription activators	Stat1	2.12							
				Stat4	1.20							
			Cytokines	Il2	2.31	2.31	2.31			2.31		
				Il4		1.39	1.39					
				Il5		2.21	2.21					
				Il6				1.79	1.79			
				Il13		2.56						
				Il21				1.70	1.70			
				Icos				1.39				
				Ifng	3.19							
			Factors inducing lineage	Cxcr5				2.27				
				Tgif1			1.56		1.56	1.56		
			Retinoic acid receptor	Rara	-1.20							
			G protein-coupled receptors	Ccr3			-9.72					
				Cxcr3	3.39		3.39					
			Beta chemokine receptor	Ccr6					2.77			
			Antigen	Cd28						1.79		
			Transcriptional repressor	Gfi1		-1.38						

Supplemental Table 24. GATA3 promotes Th subset gene expressions in Treg and Treg plasticity.

Number of genes with significant expression changes					TH1	TH2	TH9	TFH	TH17	Treg	TH22
				up	1	2	1	4	4	3	2
				down	3	3	1	2	1	4	
			group	Gene.symbol	TH1	TH2	TH9	TFH	TH17	Treg	TH22
GSE39864	Spleen and lymph nodes	Treg specific knock-out of Gata3 vs littermate controls	Transcription factor	Ahr							1.22
				Bcl6				-1.13			
				Foxo1						-1.13	
				Foxo3						-1.14	
				Gata3		1.28					
				Irf4		-1.23	-1.23	-1.23			
				Ikzf4						1.19	
				Rorc					-1.15		
				Rora					1.42		
				Tbx21	-1.55						
			transcription activators	Stat1	-1.13						
			CYtokines	Ccl17							1.26
				Il2	1.41	1.41	1.41			1.41	
				Il6				1.29	1.29		
				Il21				1.22	1.22		
				Icos				1.25			
				Ifng	-1.97						
			Factors inducing lineage	Cxcr5				1.45			
			beta chemokine receptor	Ccr6					1.49		
				Ccr8		-1.19					
			immunoglobulin	Ctla4						1.16	
			Transcriptional regulator	Bach2						-1.29	
			Antigen	Cd28						-1.17	
			transcriptional repressor	Gfi1		-1.25					

Number of genes with significant expression changes					TH1	TH2	TH9	TFH	TH17	Treg	TH22
				up	1	2	1	6	1	1	1
				down	1	1	1		1		
			group	Gene.symbol	TH1	TH2	TH9	TFH	TH17	Treg	TH22
GSE27896	Regulatory T-cells CD4+CD25+	HDAC6 knock out mice Vs. wild type mice	Transcription factor	Ahr							1.48
				Batf				1.25			
				Irf4		1.20	1.20	1.20			
				Maf				1.26			
				Rora					-1.33		
				Tbx21	-1.36						
			Transcription activators	Stat3				1.19			
				Stat4	1.21						
			Cytokines	Il4		-2.22	-2.22				
				Il21				2.46	2.46		
			Factors inducing lineage	Cxcr5				1.64			
			Beta chemokine receptor	Ccr8		1.33					
			Transcriptional regulator	Bach2						2.46	

Supplemental Table 25. BCL6 inhibits but histone deacetylase 6 (HDAC6) promotes Th subset gene expressions in Treg and Treg plasticity.

Number of genes with significant expression changes					TH1	TH2	TH9	TFH	TH17	Treg	TH22
				up	5	4	2	5	2	2	2
				down				1		1	
			group	Gene.symbol	TH1	TH2	TH9	TFH	TH17	Treg	TH22
GSE40493	Treg cell	Bcl6 knock-out Vs. wild type	Transcription factor	Ahr							1.89
				Batf				4.15			
				Foxp3						1.79	
				Gata3		3.96					
				Maf				2.68			
				Rora					1.96		
				Runx3	1.44						
				Tbx21	1.88						
			transcription activators	Stat1	2.01						
				Stat3				1.61			
			cytokines	Il13		2.32					
				Icos				3.80			
				Il10rb							
			Factors inducing lineage	Cxcr5				-1.29			1.61
				Myd88					1.77		
			Retinoic acid receptor	Rara	1.46						
			G protein-coupled receptors	Ccr3			2.38				
				Ccr4		2.80					
				Cxcr3	2.66		2.66				
			Beta chemokine receptor	Ccr8		6.57					
			immunoglobulin	Ctla4						1.90	
			Transcriptional regulator	Bach2						-1.86	

Supplemental Table 27. Bcl6 regulates Treg plasticity via following TOP 10 signaling pathways.

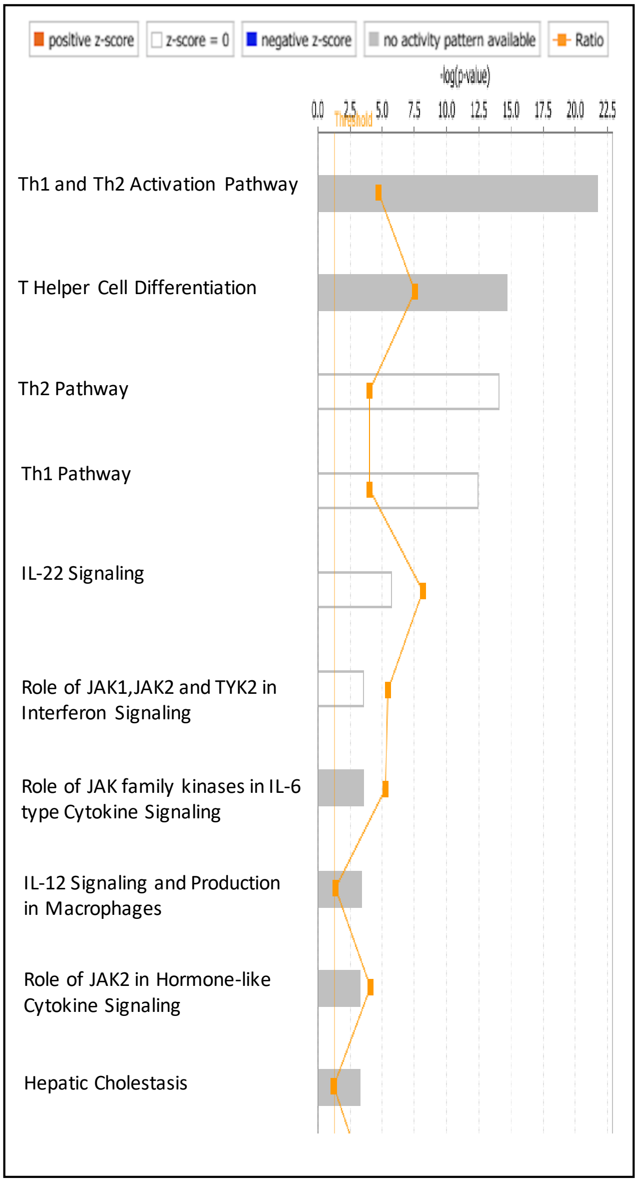

Supplemental Table 28. HDAC6 regulates Treg plasticity via following TOP 10 signaling pathways.

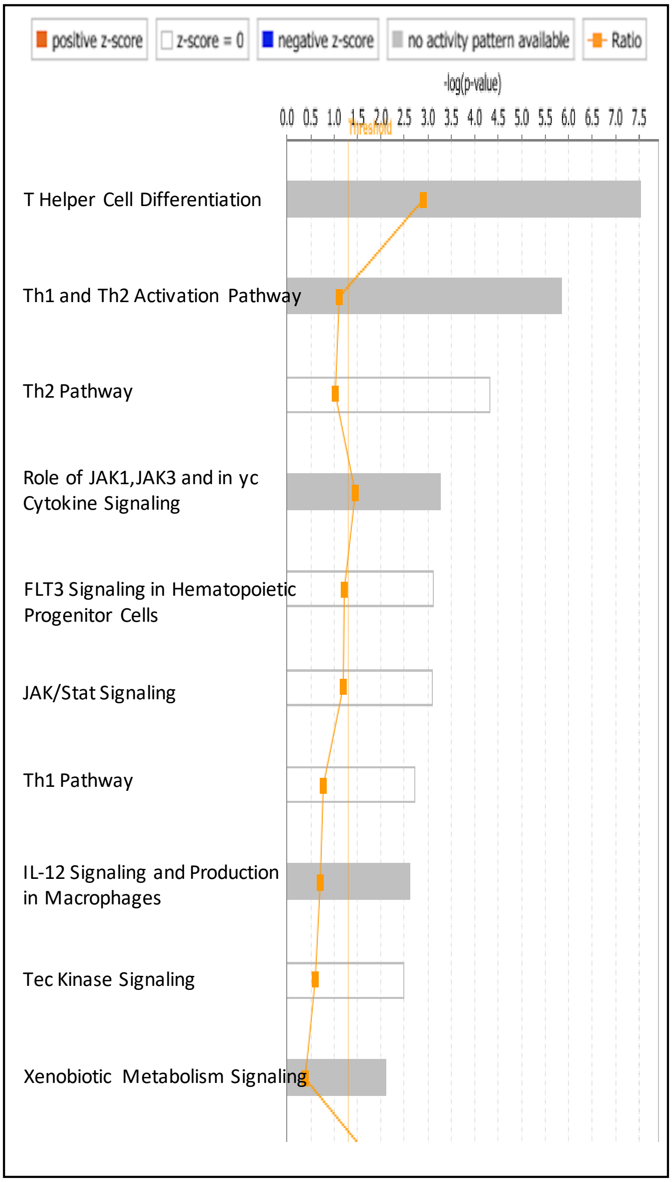

Supplemental Table 29. GATA3 deficiency up-regulates (A) and down-regulates (B) the genes via following TOP 10 signaling pathways.

A.

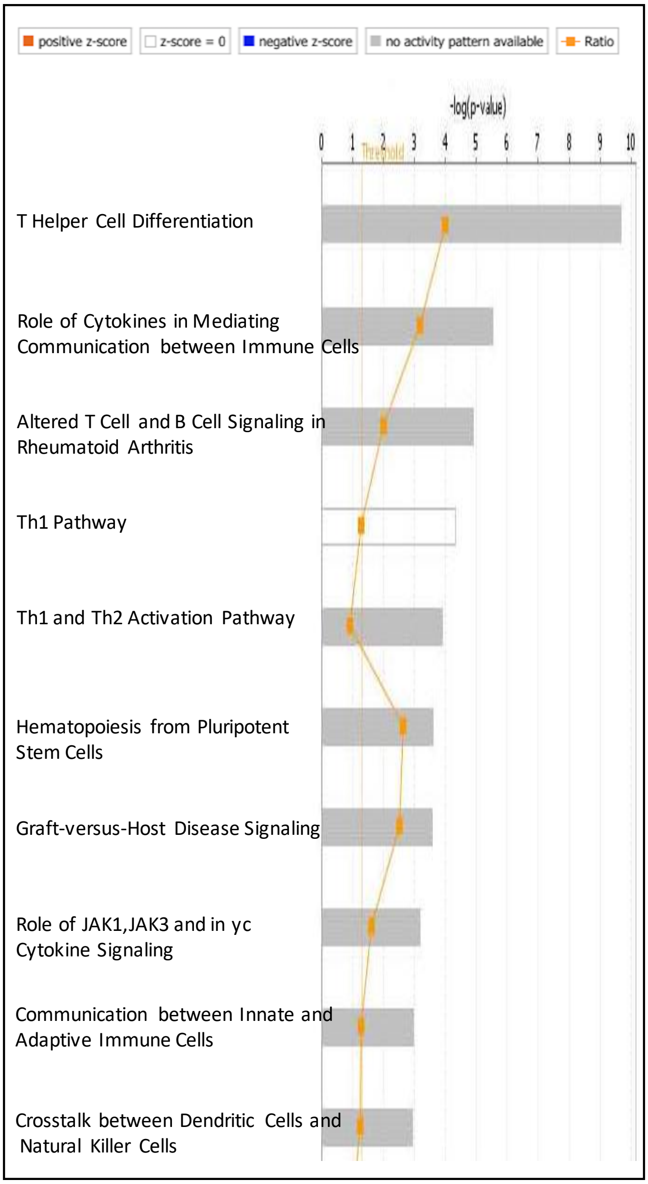

B.

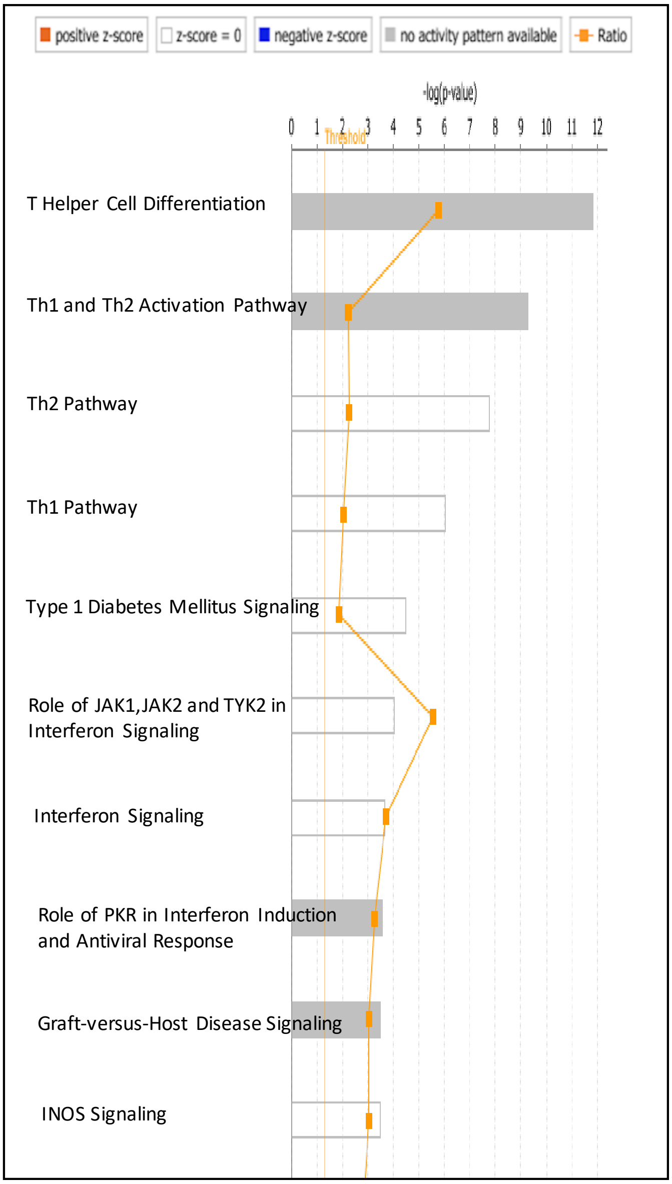

Top 10 pathways

Supplemental Table 30. Bcl6 regulates Treg plasticity for atypical APC via following TOP 10 signaling pathways.

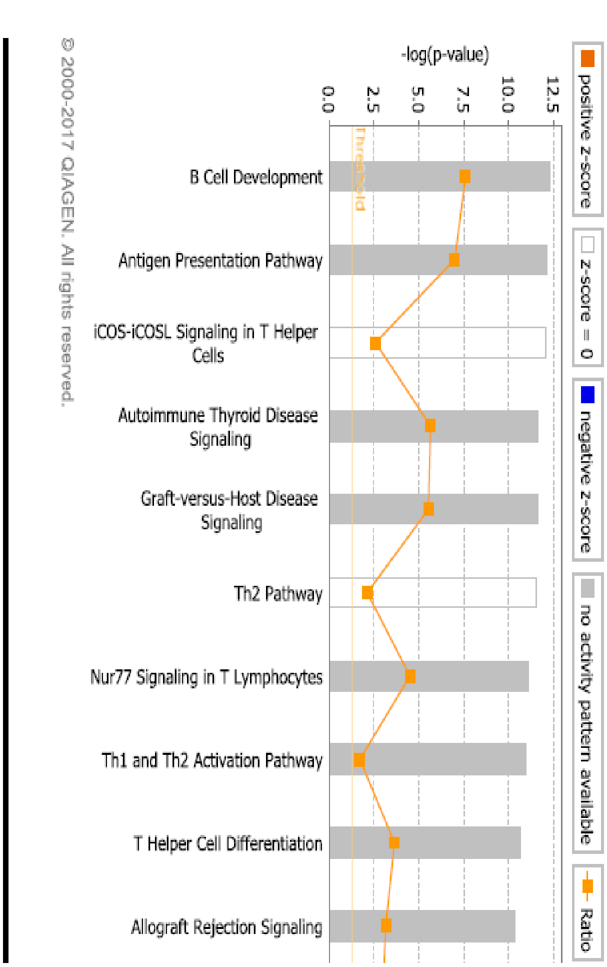

Supplemental Table 31. HDAC6 regulates Treg plasticity for atypical APC via following TOP 10 signaling pathways.

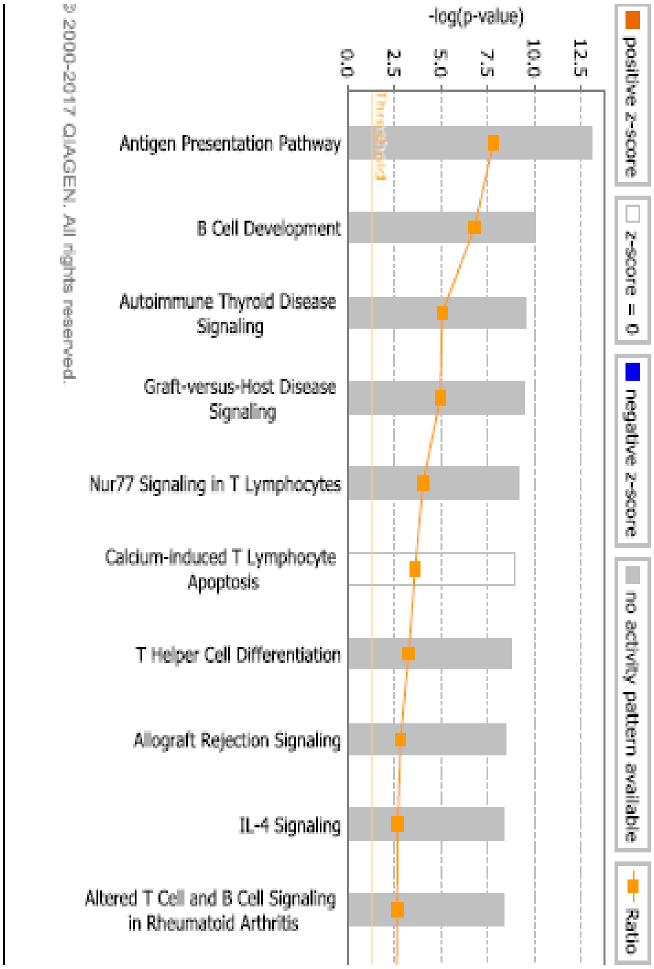

Top 10 pathways

Supplemental Table 32. Gata3 regulates Treg plasticity for atypical APC via following TOP 10 signaling pathways.

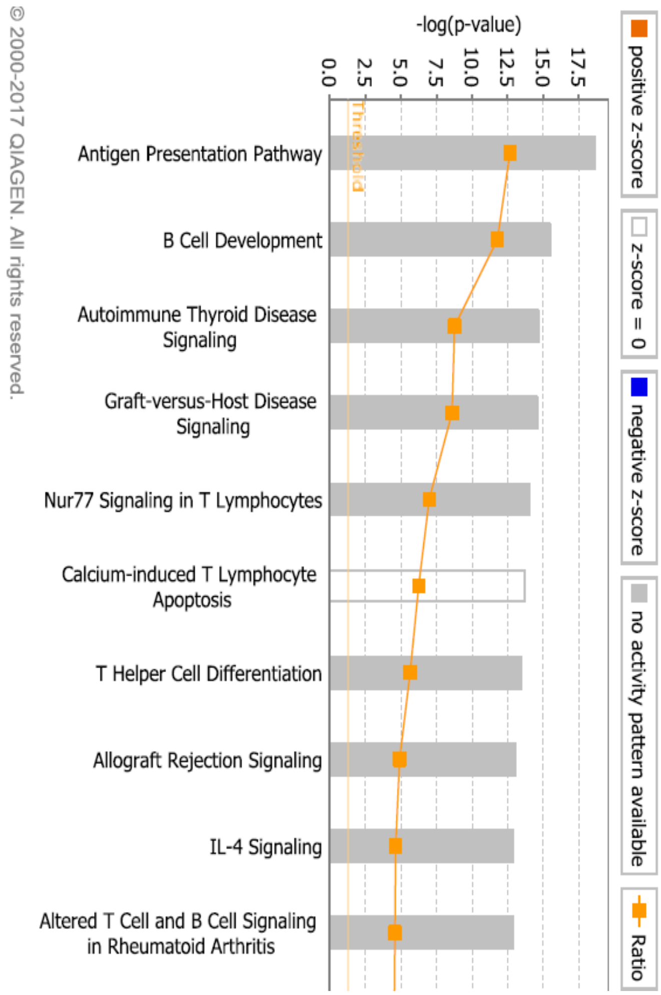

Supplement: Figure S1 — Th subset regulator mRNA transcripts are differentially expressed in healthy human tissues. [file Presentation_1.PDF]
